# Regeneration-Associated Factors in the Regulation of Adult and Post-Traumatic Neurogenesis in the Forebrain of Fish and Other Vertebrates

**DOI:** 10.3390/ijms27010247

**Published:** 2025-12-25

**Authors:** Evgeniya V. Pushchina, Eva I. Zharikova

**Affiliations:** A.V. Zhirmunsky National Scientific Center of Marine Biology, Far Eastern Branch, Russian Academy of Sciences, 690041 Vladivostok, Russia; ezharikova@imb.dvo.ru

**Keywords:** adult neurogenesis, BrdU, PCNA, adult neural stem progenitor cells, traumatic brain injury, HuCD, GFAP, glutamine synthetase, Pax genes, *Oncorhynchus masou*, *Oncorhynchus keta*

## Abstract

This review summarizes a growing collection of data on adult neurogenesis in various vertebrate species, with a focus on teleost fish and mammals. Teleost fish serve as exceptional models for studying the dynamics of the cell cycle and the functions of adult neural stem progenitor cells (aNSPCs) throughout the central nervous system (CNS). New information about the characteristics of cells in various areas of the telencephalon of non-model objects—juvenile masu salmon *Oncorhynchus masou* and chum salmon *Oncorhynchus keta*—during postembryonic ontogenesis and after traumatic injury expands the current understanding of the issue. The expression of molecular markers of adult-type glial precursors in the model zebrafish and non-model objects, juveniles *O. masou* and *O. keta*, was presented. Immunohistochemical (IHC) verification of BrdU and PCNA made it possible to identify a population of rapidly and slowly proliferating cells in the pallium of intact *O. masou* and after traumatic brain injury (TBI). In salmonids, unlike in mammals, progenitor cells are able to differentiate into neurons after injury. The expression of vimentin and GFAP in the aNSCPs has functional specificity. A comparative analysis of the expression of Pax transcription factors in various vertebrates and juveniles *O. masou* is presented. Pax genes maintain cells in an undifferentiated state and ensure the spatiotemporal formation of mature cell types in changing developing neurogenic niches. The functions of glutamine synthetase (GS) and H_2_S in the brains of vertebrates and juvenile chum salmon under intact conditions and after TBI are characterized. In fish, unlike mammals, as a result of TBI, neuronal conduction is restored in the injury area, whereas in mammals the regenerative process is complicated by neuroinflammation and culminates in the formation of a glial scar.

## 1. Introduction

Adult neurogenesis, the generation and integration of new neurons into the brains of adult organisms, is the main source of brain plasticity [1,2,3]. Neurogenesis in the adult brain occurs across all vertebrate subtypes, although its extent varies greatly depending on phylogenetic affiliation [4,5,6,7]. In mammals, adult neurogenesis is mainly limited to the dentate gyrus of the hippocampus and the olfactory bulb (OLB) and CNS [5], whereas in teleosts, adult-born neurons are generated throughout their brains [4].

Teleost fish serve as exceptional models for studying the dynamics of the cell cycle and the function of adult neural stem and progenitor cells (aNSPCs) throughout the CNS. The lifelong presence of proliferating aNSPCs in various brain niches [7,8], along with their neuroregenerative ability after brain and spinal cord injury [9], makes teleost fish extremely attractive for study. These characteristics allowed the researchers to use fish models to study the biological significance of adult neurogenesis [10], as well as brain and spinal cord repair processes [11].

Neurogenesis in adult animals is defined as a developmental process that begins with the division of aNSPCs, which generate daughter cells designed for neuronal specialization. The main difference between constitutive neurogenesis of adult vertebrates and regenerative neurogenesis, which occurs after CNS damage, is that the latter largely depends on the activation of normally dormant aNSPCs for re-entry into the cell cycle. One of the fundamental questions is which set of factors under homeostatic conditions and after injury is responsible for controlling the activity of aNSPCs.

Unlike in other vertebrates, the telencephalon of ray-finned fish develops by embryonic eversion, which represents the evagination (eversion) of the neural tube during embryogenesis [12]. As a result of this process, the pallial hemispheres are divided and surrounded by a T-shaped external ventricle. In this structure, the precursors of neurons are localized in the periventricular zone [8,13].

The brain of salmon is characterized by the preservation of embryonic structure features in adult animals: this phenomenon is called fetalization. In juvenile salmonids, this feature is characterized by the retention of a large number of neuroepithelial (NE) type cells in juveniles during the first year of life and at later stages of ontogenesis [14]. Another feature of the salmon brain is the presence of a large number of constitutive neurogenic niches located in the telencephalon, the roof of the midbrain, the cerebellum, and the brain stem [15,16]. Their functional role is currently unclear, but there are suggestions that their constituent cells become active participants in response to traumatic brain injury [17]. Similar constitutive formations containing intestinal stem cells have been identified along the trout digestive tract [18]. The analysis of early postnatal development and age-related changes in the organization of constitutive and post-traumatic processes, as well as the involvement of various regeneration-associated factors, can fill gaps in the current understanding of the development of the telencephalon of juvenile salmon, taking into account data on fetalization.

This review presents data on comparative aspects of adult neurogenesis in different vertebrate groups (Section 2) and the characteristics of neuronal stem cells in adult non-mammalian vertebrates (Section 3). Data on glial-type molecular markers of aNSPCs in the model zebrafish and non-model *O. masou* objects are presented in Section 4, information on the fate of progenitor cells in the neurogenic zones of mammals and fish is summarized in Section 5, data on cell migration in the telencephalon of zebrafish and *O. masou* are presented in Section 6. The features of the differentiation of precursors in comparative aspects in mammals, zebrafish, and *O. masou* are presented in Section 7. The characteristics of adult-type precursors in zebrafish and *O. masou* are given in Section 8, and information on the immunolocalization of molecular markers GFAP and vimentin in the telencephalon of fish is summarized in Section 9. The participation of PAX family transcription factors in CNS development and neuroregeneration is considered in Section 10, the functions of glutamine synthetase are discussed in Section 11, and the participation of hydrogen sulfide in CNS functions in fish and mammalian models is summarized in Section 12. Conclusions and future prospects are presented in Section 13 of this review.

## 2. Adult Neurogenesis in Vertebrates

Neurogenesis in the postembryonic period is observed in most vertebrates. However, most of the data in this area were mainly obtained on two mammalian stem cell models: the subventricular zone (SVZ) of the lateral wall of the lateral ventricle and the subgranular zone (SGZ) of the hippocampus, where slowly proliferating astrocyte progenitor cells give rise to new neurons [19,20]. The differentiation process in the SVZ is preceded by the active migration of progenitor cells into the olfactory bulb (OLB) along the rostral migration stream (RMS). In the OLB, such neuroblasts differentiate mainly into GABA-ergic interneurons [20].

Many aspects of adult neural stem and progenitor cell (aNSPCs) biology remain unexplored. For example, the origin of neurogenic “niches” [21] and the influence of various cellular factors on stem cells, which determine whether they remain in a stem cell state or begin to differentiate, are not yet fully understood.

**Figure 1:**
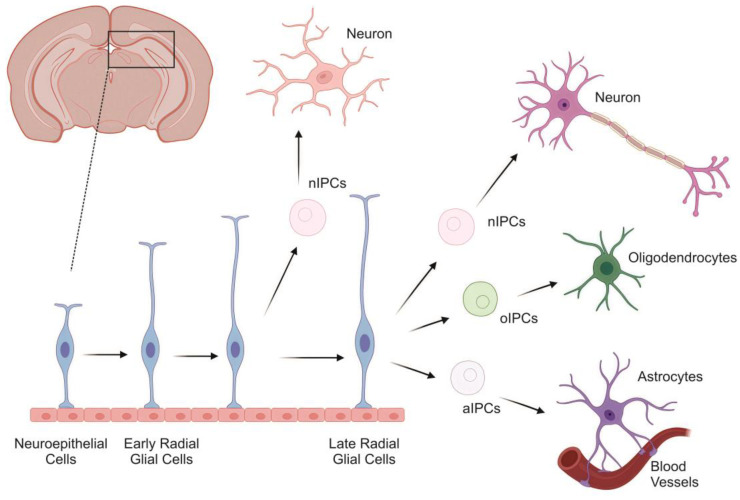
Diagram of the development of neurons and glial cells in adult mammals. Neuroepithelial cells of the periventricular zone of the I and II cerebral ventricles give rise to early radial glial cells, which can then differentiate into neurons or give rise to intermediate progenitor cells (nIPCs). During proliferation, early radial glia differentiate into late radial glia, which are capable of directly producing neurons or producing three types of precursors: astrocytic (aIPCs), oligodendrocytic (oIPCs) and neuronal (nIPCs). These precursors further differentiate into astrocytes, oligodendrocytes, and neurons.

One of the transcription factors [22,23] that affects stem cells in both the SVZ and SGZ is Sox2 [24,25]. Another transcription factor, Olig2, triggers cell determination in the SVZ and RMS, while Pax6 stimulates neuroblasts to differentiate into TH-positive neurons in the OLB [26,27]. Neurogenesis in adult mammals is limited to the SVZ and SGZ (Figure 1), but is much more widespread in other vertebrates: reptiles [26], birds [27], and fish [28]. These models can provide insight into the mechanisms of differentiation of other neuronal subtypes in the adult brain, other than OLB neurons or hippocampal interneurons. Such studies may help identify common features of adult neurogenesis in mammals and identify the main factors supporting stem cells. In the telencephalon of fish, proliferation and neurogenesis continue throughout life, correlating with prolonged brain growth and high regenerative capacity [28]. Limited proliferation areas suggest the presence of neurogenic niches. In the adult brain of *D. rerio*, new neurons are formed in the OLB, the telencephalon, the hypothalamus, the preoptic region and the optic tectum, as well as in the cerebellum [7,29]. However, the location and molecular characteristics of the aNSPCs remain unclear. Progenitor cells in the telencephalon of *D. rerio* are located in both the dorsal and ventral regions and produce a unique combination of molecular markers. Neurogenesis in the brain in *D. rerio* and in mammals has many common features, which makes it possible to consider *D. rerio* a good model for studying neurogenesis [30].

Neurogenesis in adult animals is significantly more pronounced in birds, reptiles, amphibians, and fish than in mammals. This process is usually localized in several periventricular regions of the brain and performs adaptive functions. However, the areas of the brain where proliferative zones are located depend on their functional specialization [31,32] and vary from species to species. Currently, there is a significant lack of detailed information for a comparative assessment of species-specific differences and for determining the overall significance of such observations for each systematic group.

For the first time, studies of adult neurogenesis were conducted in songbirds [33,34]. Some of the available results have been obtained in animals in their natural habitat, proving that neurogenesis in the adult brain is not exclusively a laboratory observation. Neurogenesis is also observed in adult reptiles, such as lizards, turtles, and amphibians [35]. Adult neurogenesis has been studied in several teleost fish: stickleback [36], guppy [37] *Lebistes*, goldfish [38], trout *Salmo trutta fario* [39], medaka *Oryzias latipes* [39]. However, these studies have not yet provided data on specific cell types (their phenotypes) involved in adult neurogenesis. Recent studies have established the features of this process in *D. rerio*, which has significant differences from other fish species [7,40]. These studies allow for a more comprehensive comparison of the organization of neurogenic zones, as well as an investigation of the survival and integration of adult-born neurons into neural networks.

In all animal species studied to date, neuronal proliferation occurs throughout life. In birds, proliferation zones are most often located along the walls of the lateral ventricle of the telencephalon, and produce new neurons in the dorsal nuclei of the telencephalon in the region of the nidopallium (which is the highest vocal center), the hippocampus, and striatum structures (the paraolfactory lobe containing the nucleus of the *vagus nervus*). A fundamental difference is observed in bony fish, where adult neurogenesis occurs in all regions of the brain [7]. However, as in other vertebrates, it is concentrated at specific local points, indicating the existence of localized regulatory mechanisms governing neurogenesis.

## 3. Neuronal Stem Cells in Adult Non-Mammalian Vertebrates

The sources of new neurons in adult mammals are aNSCs, and it is important to find out whether these cells have similar characteristics to the precursors of neurons in non-mammals. Mature NSCs should have two main properties: multipotency and the ability to self-renew [41]. NSCs have the ability to produce neurons and glia, both in vivo and in vitro, although differentiation into a neuron or glial cell may be determined by conditions of the cellular microenvironment and other dynamic factors [42]. The second property means that during division, a cell either gives rise to two stem cells, or generates a stem cell and a cell that subsequently differentiates into a neuron or gliocyte [7]. Theoretically, cell self-renewal is not predetermined. Moreover, one of the basic characteristics of stem cells is being at rest, meaning the division process is relatively rare.

Although the definition of neural stem cells (NSCs) is relatively straightforward, demonstrating their properties in practice is challenging. In particular, the ability to proliferate and multipotency of cells can only be tested by performing repeated sequential transplantations of single cells and subsequent depletion of the entire cell population, as has been established for the hematopoietic system [43]. Therefore, accurate identification of endogenous adult NSCs and verification by existing methods are currently difficult. In practice, “long-term”, “self-renewing” or “slowly proliferating” cell populations are usually identified using BrdU labeling [44].

Following the discovery of neurogenesis in the adult vertebrate brain, a key question has been which cells can be classified as NSCs according to classical criteria. Equally important is determining which of these criteria are unequivocally met by mammalian NSCs. Since the first evidence of neurogenesis in the brains of adult rodents was published about 40 years ago, the toolkit for detecting and studying NSCs has expanded significantly [45,46]. Contradictory results have been obtained in studies on mice, suggesting that the ependymocytes lining the walls of the lateral ventricle are stem cells [47].

The prevailing hypothesis currently holds that astrocytes expressing glial fibrillary acidic protein (GFAP) are the main source of new neurons. However, in further experiments, this hypothesis was not confirmed, and it was found that astrocytes form a heterogeneous population, with only some of them being stem cells. The existence of slowly proliferating astrocytes was established using pharmacological drugs that reduce the rate of proliferation and labeling of BrdU. For example, Ara-C kills proliferating cells without affecting the dormant cell population [48]. Thus, BrdU labeling in combination with Ara-C makes it possible to identify slowly proliferating cells.

One of the morphological characteristics of ependymocytes is the presence of a single cilium in contact with the lumen of the cerebral ventricle [48]. Three cell populations have been identified in the subventricular zone. The slowly dividing B cells, detected by electron microscopy, are astrocytes connected to the ventricle through the ependyma. They give rise to a population of more rapidly proliferating C cells, which then produce A cells, the migrating neuroblasts [48]. Based on the application of retroviral techniques in the subgranular zone, using Ara-C and electron microscopy data, primary precursors were identified as a radial or horizontal population of slowly dividing astrocytes. They lead to the proliferation of D1 cells, which generate young neurons [49].

## 4. Molecular Markers of Glial-Type aNSPCs

Immunolabeling of GFAP and another glial cell marker, brain lipid-binding protein (BLBP), revealed the presence of radial glial cells along the entire brain of *D. rerio*. This population of slowly proliferating cells was also detected in vivo by labeling with BrdU and proliferating cell nuclear antigen (PCNA) [13]. This experiment was conducted to distinguish populations of rapidly proliferating (BrdU+/PCNA+) from slowly proliferating (BrdU−/PCNA+) cells. The principle of the experiment is that PCNA should be present in all phases of the cell cycle (except when cells remain in the G1 phase for a very long period, leading to PCNA degradation by p21 cell cycle inhibitor [50]) while BrdU is included only in S-phase. In general, if the cell cycle is short, a larger number of cells will remain in the S-phase, and if the cell cycle is long, the number of BrdU-labeled cells will be smaller. Based on this paradigm, as well as on the fact that the BrdU label is preserved, *D. rerio* have identified cells with a long cell cycle in the telencephalon, pineal pedicle, hypothalamus, cerebellum, midbrain roof, and isthmus region [8,15].

**Figure 2:**
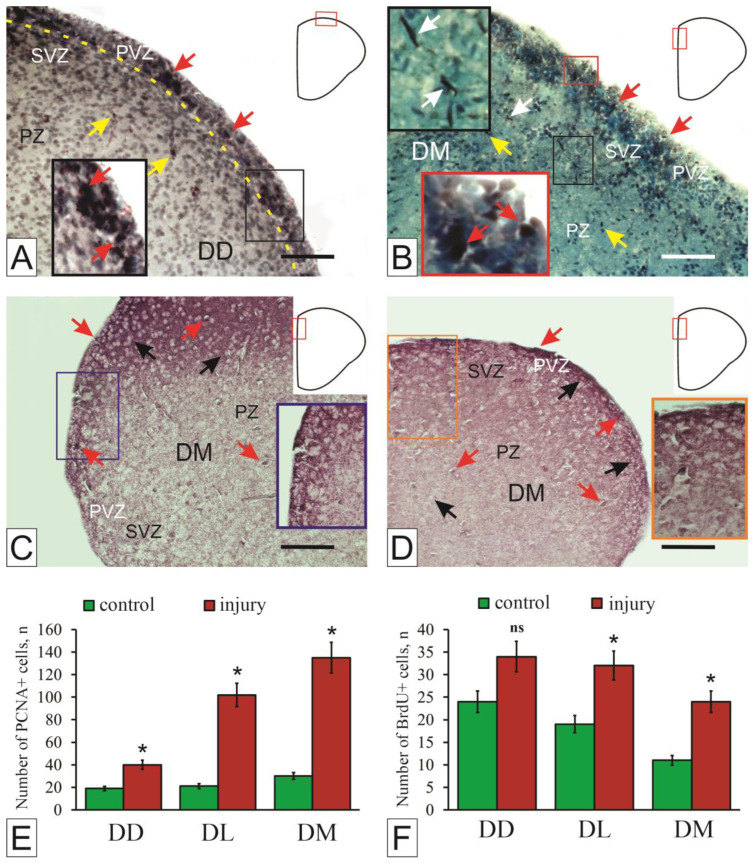
Proliferating cells nuclear antigen (PCNA) distribution (**A**,**B**,**E**) and BrdU of immunopositive cells (C,**D**,**F**) (representative) in the dorsal region of the telencephalon of juvenile masu salmon (*O. masou*) original image from [49,50]. (**A**,**C**)—intact animal; (**B**,**D**)—after TBI; (**E**,**F**)—the ratio of immunopositive cells in the control (intact animals) and after TBI, ns–non significant (n = 5; * *p* < 0.05—compared with control). (**A**)—dorsal zone (DD), (**B**)—medial zone (DM). Black and red rectangles outline the insets in the figures, yellow dotted lines show the boundaries of the ventricular zone (PVZ), subventricular zone (SVZ), parenchyma (PP) red arrows show PCNA+ cells in PVZ and SVZ, yellow–PCNA+ cells in PP; and white–migrating cells. (**C**,**D**)—medial zone (DM), colored rectangles outline the insets in the figures, red arrows show BrdU+ cells, black–immunonegative cells. Scale bar: 100 µm.

In juvenile *O. masou*, PCNA immunolocalization was detected in the dorsal, lateral, and medial parts of the telencephalon [51]. The topography of the distribution of PCNA+ cells in each zone had similarities: in the surface and subventricular layers, single or proliferating cells forming small clusters were identified (Figure 2A). In intact animals, a development of the pronounced surface periventricular layer containing PCNA+ cells was noted, which extended throughout all areas of the dorsal region (Figure 2A).

Three days after TBI, the telencephalon of *O. masou* showed significant changes in the proliferative activity of cells, both in the proliferative zones and in the parenchyma of the telencephalon (Figure 2B,D–F). The most significant changes in the topography of proliferating cells were associated with the restructuring of a single proliferative superficially located periventricular layer and the formation of local zones of induced neurogenesis [51]. At the same time, the periventricular proliferative zone (PVZ) was generally not verified (Figure 2B). The formation of local clusters of cells, PCNA+ elements, and extensive populations of migrating cells was observed (Figure 2B). These changes occurred after the traumatic injury and can be classified as reparative rearrangements [50].

A study of the pallium of intact juvenile *O. masou* [51] revealed a heterogeneous population of BrdU+ cells and nuclei. BrdU immunolabeling was detected in the dorsal telencephalon [52]. According to Traniello’s classification [53], intensively labeled cells and nuclei were isolated from BrdU-immunolabeled elements (Figure 2C). The results of BrdU immunolabeling are consistent with data from PCNA immunolabeling in the pallium of zebrafish [54] and juvenile *O. masou* [51]. PCNA labels an additional DNA polymerase delta, which remains in the cell for 24 h after the completion of mitosis [55], but the level of PCNA activity decreases by 30% [55]. BrdU immunolabeling makes it possible to identify cells and nuclei in the S-phase of the cell cycle, whereas PCNA labeling can help visualize a larger population that includes cells actively proliferating as well as those that have recently exited the cell cycle [56]. Patterns of tangential surface migration of elongated BrdU+ cells were detected in the lateral pallium (Figure 2C), and radial migration of BrdU+ cells from the PVZ to the SVZ was recorded in the medial pallium [51]. Within the proliferating cell population of the pallium, most dorsal cells were in the S phase, while others were at different stages of the mitotic cycle, including those migrating toward the medial pallium (Figure 2E). Both PCNA labeling and experimental administration of BrdU revealed immunopositive cells and nuclei in juvenile *O. masou* as part of the SVZ and PVZ (Figure 2A,C). However, whereas BrdU labeling identified only a few individual cells in the PVZ and SVZ, PCNA immunolabeling revealed a much larger number of cells with proliferative activity.

Studies on *D. rerio* indicate the existence of factors that keep stem cells in the ventricles [54]. BrdU-labeled cells remain in the periventricular region for several weeks. Because such cells were found in two-year-old individuals, they probably persist at a later age. These data indicate that some of the progenitor cells in the adult telencephalon of *D. rerio* are a slowly proliferating and self-renewing population. It should be noted that these label-preserving cells express glial factors similar to those of mammalian NSCs [56]. Together with some characteristics of molecular expression (for example, SOX2), these data indicate that the ventricles of the *D. rerio* telencephalon contain progenitor cells with the properties of NSCs. It is important to note that such cells in *D. rerio* line the entire ventricle in the dorso-ventral direction, unlike in mammals.

Interestingly, progenitor cells in adult fish differ significantly from their embryonic counterparts. For example, one of the distinguishing features is the acquisition of glial cell characteristics, which appear only at the end of the embryonic period [57]. In addition, the elements necessary for the expression of *ngn1* in aNSPCs differ from those regulating expression at the embryonic stage [58]. The expression of genes in adulthood and the embryonic period differs significantly. For example, *emx3* and *ngn1* are expressed in the dorsal region of embryos, while *dlx*, *ash1*, and *olig2* are expressed in the ventral region [59]. The expression of the transcription factor *Pax6a* is limited to the ventral telencephalon, and *Pax6b* is completely absent within 24 h after fertilization [59]. This differs significantly from the expression pattern of these transcription factors in adult fish, where most of these genes are expressed throughout the dorso-ventral axis. It remains unclear whether these changes result from cell migration or differential transcriptional regulation, but they suggest a unique set of progenitor cells in neurogenic niches in adults, both as *D. rerio* to refer to mice.

Further research will help better understand distinctive features of embryonic and adult progenitor cells and elucidate the mechanisms regulating the functional activity of adult NSCs. Evidence for the process of self-renewal of nerve cells in mammals is based on the so-called “analysis of neurospheres” [60]. Dissociated cells from the SGZ or SVZ are cultured in vitro in the presence of growth factors (usually epidermal growth factor or fibroblast growth factor), which induce the cells to form spherical clusters. Subsequently, cells from the secondary neurospheres, obtained from single cells of the primary neurosphere, are analyzed. Although this method reveals the potential of isolated cells, it does not fully reflect the essence of self-renewal processes. For example, it is not clear how cells in the neurosphere form transitional progenitors. This issue is especially relevant for the SGZ, where the ability of cells to generate secondary neurospheres remains controversial [61]. Moreover, increasing evidence suggests that cells in the neurospheres behave differently than in vivo. For example, in cell culture studies, combinations of cellular markers have been found that have never been found in vivo. In addition, even parameters such as the density of cell distribution can affect the state of proliferation and/or cell viability. Finally, in vitro cultivation can alter the genetic and epigenetic properties of cells [61].

In vivo, the indefinite self-renewal of NSCs in mammals can also be questioned. A decrease in the level of cell proliferation associated with age (as shown by BrdU labeling) and reduced survival of newborn neurons are observed in both the SGZ [62] and SVZ [63]. Presumably, NSCs do not actually self-renew, unlike, for example, hematopoietic stem cells. Whether this phenomenon results from changes in the intrinsic properties of stem cells or from the absence of external influence by the neurogenic niche remains to be determined by future studies.

## 5. The Fate of Progenitor Cells in the Neurogenic Areas of the Mammalian and Fish Brains

The potential fates of the progenitor cells of the SGZ and SVZ are often traced in vitro using neurospheres, because they contain not only the stem cells themselves, but also their already differentiated descendants. However, this method has significant limitations.

NSCs in the SGZ and SVZ mainly produce new neurons. Glial renewal is somewhat limited, although oligodendrocytes can originate either from cells of the SVZ or by glial differentiation of progenitor cells as part of the rostral migration stream [64]. Cell proliferation was observed in in vivo studies after injection of retroviruses into the brain ventricles [65]. However, this method is limited by two factors. Firstly, due to the small time window, similar to BrdU, the virus integrates into the genome of only rapidly proliferating cell populations, and does not affect slowly proliferating stem cells. Secondly, according to statistics, the virus integrates into only one of the two daughter cells. Lentiviruses, in contrast, can be incorporated into dormant cells and, therefore, are suitable for use in tracking descendants of subgranular and subventricular stem cells [66]. At the same time, the multipotency of a single infected cell makes it possible to make better use of neurosphere analysis during dissociation and culture cultivation of lentivirus-infected progenitor zones [66].

In teleost fish, neurospheres derived from the telencephalon and cerebellum of *Apteronotus leptorhynchus* can differentiate into neurons as well as GFAP-positive or vimentin-positive glial cells [67]. However, the limitations that exist in the interpretation of neurosphere research data are also valid in this case. In order to determine the multipotency of progenitor cells in vivo, it is important to study them without replanting them into culture. In *D. rerio*, as in mammals, progenitor cells mainly form neurons, not glial cells. In previous experiments, several new non-neuronal cells were described in the outer layer of the OLB and in the parenchyma of the telencephalon [9]. In addition, a small number of newly formed cells expressing the S100 astrocyte marker were found along the ventricle of the telencephalon, in the dorsal hypothalamus, and in the cerebellum [13]. The reasons for this pattern of distribution of neurons and non-neuronal cells in *Danio rerio* are not yet clear.

However, the limited production of astrocytes in the adult brain is consistent with their overall low content in the brains of teleost fish. All these observations indicate comparable properties of progenitor cells, which give rise to most new neurons in the brains of adult bony fish and mammals. Given the visible progress in the study of NSCs identification, the current definitions of NSCs in both taxa can be somewhat expanded. For example, the possibility of unlimited self-renewal is not supported in in vivo experiments. This also suggests that further analyses of the multipotency of an individual progenitor cell are required. Sites with pronounced proliferative activity have been described in the adult brain of teleost fish [37,68], including the retina of *D. rerio* [30], the OLB, the telencephalon and cerebellum [7], as well as the posterior brainstem and spinal cord [30]. However, with the exception of the cerebellum, it has not been established which cell pools give rise to neurons and glia in different brain regions [8].

To study these issues, BrdU labeling was used, and the fate of labeled cells was monitored. According to previous observations, many proliferating cells were found in the brain of adult *D. rerio* and 16 sites with pronounced proliferative activity were identified [8]. The telencephalon of *D. rerio* is usually divided into a dorsally located pallium and a basally located subpallium. The pallium is further divided into dorsal, ventral, lateral, and medial regions, and the subpallium is divided into dorsal and ventral regions [69]. In the telencephalon, the ventral subpallium is intensely labeled and appears as a longitudinal band of BrdU-positive cells connecting the posterior part of the telencephalon with the OLB. No BrdU labeling was observed inside the OLB itself, but several positive glial cells were found in the surface layer of the olfactory nerve [70]. Most proliferating cells were located near the ventricle. These cells form an extended region on the dorsal outer surface of the telencephalon, which corresponds to the inverted type of telencephalon formation in teleost fish [71]. In addition, a zone with active cell proliferation was found at the junction between the pallium and the ventral region of the telencephalon, which overlaps the optic tract. Several positive cells were also detected in the parenchyma. A study of BrdU-labeled cell distribution has shown that, despite their presence at all levels of the dorso-ventral region, their number can vary significantly. The ventral subpallium had the highest density of BrdU-positive cells, whereas the dorsal subpallium had four times fewer cells, and the medial and dorsal pallium had ten times fewer cells. These observations indicate that the ventral subpallium either contains a large pool of proliferating cells or exhibits a higher cell division rate than the dorsally located areas.

To solve this problem, the BrdU labeling index of each region was estimated by calculating the number of BrdU-labeled cells (number of cells in the S-phase) within an actively dividing population expressing PCNA [55]. In addition to the data on a higher density of PCNA-positive cells located along the ventricle in the ventral subpallium, the number of double-labeled BrdU/PCNA cells in this region doubled. Most progenitor cells in the ventral subpallium proliferated rapidly, whereas progenitor cells from other regions proliferated more slowly. Consistent with a delayed cell cycle in the dorsal subpallium, medial, dorsal, and lateral pallium, the number of cells with BrdU inclusions in these areas steadily increased during the 10 days of the experiment. Thus, it is obvious that the proliferating cells of the telencephalon are represented by several subpopulations that differ in their rates of division.

The proliferative activity and subsequent fate of adult neural stem cells vary depending on their localization in different areas of the brain. Currently, the mechanisms of this remain poorly understood. Given that these properties have significant interspecific differences, comparing different models helps uncover the specific mechanisms responsible for cell proliferation and differentiation. At the level of a single brain region, what limits the fate of particular NSC groups across species remains unclear.

For example, the subpallial progenitor zone in *D. rerio*, which produces olfactory bulb neurons, also produces GABA- and TH-positive telencephalic cells [13]. This phenomenon has never been observed in mice, where the SVZ produces only the neurons of the OLB. If we look at this problem more globally, at the level of the entire brain, it becomes clear which mechanisms and molecular components contribute to active neurogenesis in some species, and why they do not work in others. For example, in *D. rerio*, long-lived progenitor cells located along the dorsal surface of the ventricles of the telencephalon generate new neurons [13]. In mice, neurogenesis does not occur in the cerebral cortex. Elucidating the mechanisms of neurogenesis in the telencephalon of *D. rerio* will help identify what is missing, or, conversely, what should be present in the adult mammalian brain for the formation of new neurons. This rationale is also valid for other areas where adult neurogenesis has been found in *D. rerio*, for example, in the hindbrain or hypothalamus, but not in equivalent structures of the adult mammalian brain. Finally, by comparing animal models with different lifestyles, behavioral patterns, and living in different environmental conditions, we can achieve a deeper understanding of the role of adult neurogenesis.

## 6. Cell Migration in the Adult Telencephalon

Studies using BrdU labeling at various time intervals (3 days and 2, 3, 4, and 8 weeks after injection) showed that the progeny of proliferating cells are located in close proximity to the brain ventricle. Similar experiments also make it possible to track cell migration. Three days after BrdU labeling, no major changes were observed in the telencephalon, but several BrdU-labeled cells were detected in the OLB. More noticeable changes were observed after 2 weeks. During this period, numerous positive cells were found in OLB. The olfactory bulb in *D. rerio* consists of three layers (olfactory nerve, glomerular layer, and inner layer) [72]. After 2 weeks, BrdU-positive cells were predominantly found in the basal layer, and after 4 and 8 weeks they were also detected in cortical layer. Thus, cells produced outside the OLB migrate there within 2 weeks. At the same time, the total number of positive cells in the ventral subpallium decreased, and most of the cells that retained the BrdU labeling formed clusters along the ventricle. In addition, cells located along the ventricle in the dorsal subpallium and pallium migrated to the parenchyma two weeks after BrdU labeling [73]. Cell migration continued for a long time, and persisted 19 days after labeling. The results of the study indicate that within two weeks, proliferating cells from the ventricles moved to the anterior region of the ventral subpallium, OLB and parenchyma.

Doublecortin (DC) is expressed by newly created and migrating neurons in the intact pallium of *O. masou*, localized both in the cytoplasm and in cell nuclei; its presence ensures many intracellular processes [51]. DC expression may be important during axonal growth and/or synaptogenesis in the adult body [74], as well as in the dendrite growth cone [75]. DC expression is conserved in post-mitotic neurons and coincides with calretinin expression [75].

**Figure 3:**
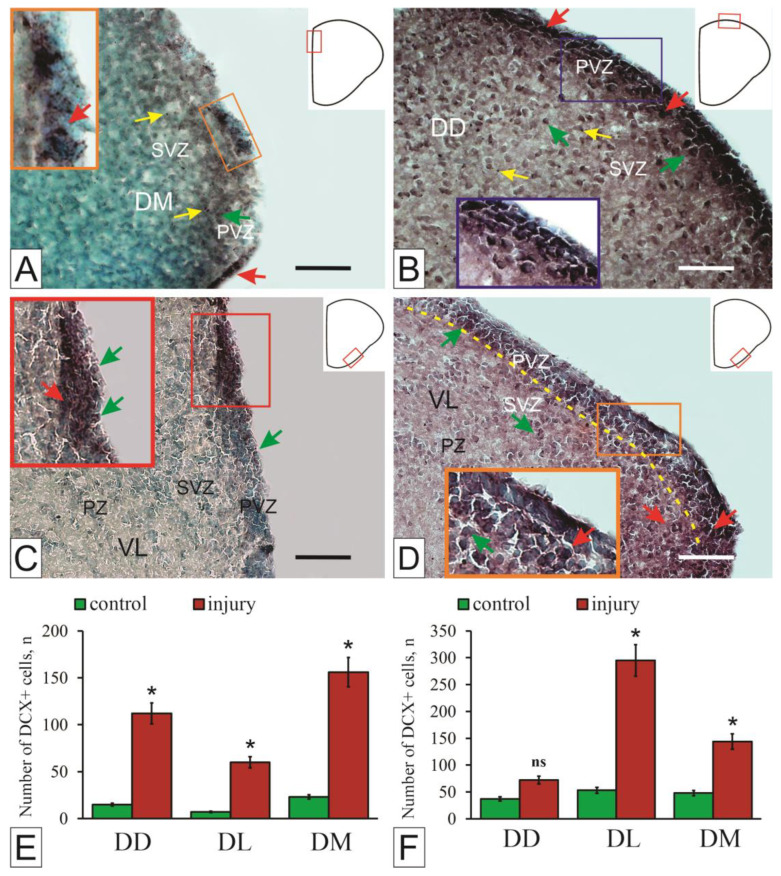
Doublecortin distribution (representative) in the pallial (**A**,**B**,**E**) and subpallial (**C**,**D**,**F**) proliferative zones of the telencephalon of juvenile *O. masou* original image [15,50]. (**A**,**B**)—in intact animals; (**C**,**D**)—after TBI; (**A**)—medial (DM), (**B**)—dorsal (DD) zones of the pallium; rectangles outline the insets in the figures, red arrows show intensely labeled cells, green arrows—moderately labeled, yellow—immunolabeled extracellular granules. (**C**,**D**)—lateral area (VL) of the subpallium; rectangles outline the insets in the figures, the red arrows show intensively labeled cells, the green arrows show moderately labeled cells, the yellow dotted line indicates a layer of labeled cells in the proliferative zone, the perventricular zone (PVZ), the subventricular zone (SVZ), and the parenchyma (PZ). (**E**,**F**)—the ratio of immunopositive cells in the control (intact animals) and after TBI, ns–non significant (n = 5; * *p* < 0.05—compared with the control). Scale bar: 100 µm.

In studies in the pallial region of the telencephalon of juvenile *O. masou*, distinct intensely DC+ cells and DC+ granules were detected (Figure 3A,D). A study of DC localization in the pallium of *O. masou* showed low protein expression in cells of primary neurogenic zones [51]. Compared with other intermediate filament proteins, vimentin and GFAP, the number of DC+ cells is reduced in the pallium PVZ, which probably indicates a relatively low content of postmitotic neuroblasts in the PVZ. Nevertheless, different levels of DC+ granules were found in different areas of the intact pallium (Figure 3A,D). The expression of DC in the form of granules in the pallium of *O. masou* may be necessary to ensure the neuronal plasticity of adult neurons [51].

The results of studies on juvenile *O. masou* are consistent with data obtained on birds and other vertebrates [51]. The presence of DC-positive cells in the pallial area (Figure 3A) is probably associated with constitutive postembryonic neurogenesis and neuronal plasticity, which is consistent with the results of studies on pallium of *D. rerio* [8] and *Nothobranchius furzeri* [76]. Studies on juvenile *O. masou* confirm the simultaneous process of radial migration of newborn cells from the proliferation zone to the surrounding subpallial cell masses (Figure 3C), which corresponds to previously obtained data on other fish species [76].

As a result of traumatic injury, the immunolabeling of DC in the telencephalon of juvenile *O. masou* [16,51] underwent significant changes (Figure 3B,D–F). Currently, there are relatively few studies related to the use of DC as a marker of reparative neurogenesis in the mammalian brain following CNS injury [77]. Compared with intact animals, intensive DC labeling of cells was observed in the periventricular regions of the pallial and subpallial areas of the *O. masou* brain (Figure 3A,B,D,E).

A characteristic feature revealed by DC labeling in the pallium of a *O. masou* after injury is a pronounced pattern of cell migration from the neurogenic zone of the pallium deep into the parenchyma of the brain (Figure 3B). This is confirmed by the simultaneous process of radial migration of numerous neuroblasts newly formed as a result of the traumatic injury [51]. The origin of cells migrating to OLB was previously unknown [8]. BrdU-positive cells were not detected in the OLB immediately after labeling; therefore, new neurons must appear elsewhere. Additionally, the number of BrdU-positive cells in the ventral subpallium decreases over time, while their number in the central nervous system increases.

These observations indicate that OLB neurons may originate from a population of proliferating cells located in the ventricular wall in the ventral subpallium. In order to confirm this assumption, attempts were made to identify the migration path and characteristics of cells moving towards OLB. Unlike in mammals, only small numbers of GFAP-positive cells were detected in adult *D. rerio*, which is consistent with recent studies of radial cells in the adult brain of this species using the AroB marker [78].

However, intensive PSA-NCAM staining has been identified in the subpallium adjacent to the OLB in *Danio rerio*, similar to the migration of PSA-NCAM-labeled neuroblasts in mammals [79,80]. These data suggest that the band in the ventral subpallium of *D. rerio* may correspond to the migration flow of neurons and neuroblasts moving from the telencephalon to the OLB, analogous to the RMS in the mammalian brain.

## 7. Differentiation of Progenitor Cells

In order to determine whether adult neurogenesis contributes to an increase in the population of GABA-ergic neurons in OLB [81], as it occurs in mammals, the expression of the GABA-synthesizing enzyme GAD67 in BrdU-positive cells was analyzed [82]. Four weeks after labeling, a large proportion of BrdU-positive cells in OLB expressed GAD67, which indicates an increase in the number of GABA-ergic neurons in the OL of *D. rerio.* Small percentage of BrdU-positive cells express tyrosine hydroxylase (TH) 4 or 8 weeks after labeling [82]; this accounts for less than 5% of the total number of TH-positive neurons, confirming similar data in mammals [83].

It is likely that these neurons are also GAD67 positive, since, as in rodents, TH-positive neurons can produce GABA in the OLB of *D. rerio* [83,84]. Taken together, these results show that neurogenesis in adult *D. rerio* promotes the generation of several types of OLB neurons: GABA-ergic interneurons in inner layer and TH-positive interneurons in the cortical layer, which is comparable to that in mammals.

HuCD is a calcium-binding protein and a member of the HuECD family. Hu proteins are RNA-binding proteins similar to ELAV, named after the *Drosophila* ELAV protein in which they were first discovered. Four types: HuC, HuB, HuD, and HuR with a molecular weight of about 40 kDa have been found in humans. Three of them, HuC, HuB, and HuD, are expressed in neurons [85]. The role of HuCD in neurogenesis is well established; these proteins influence neuronal differentiation during both constitutive and reparative neurogenesis [50]. The survival of newly formed nerve clusters is necessary for the healing of brain tissues [86].

**Figure 4:**
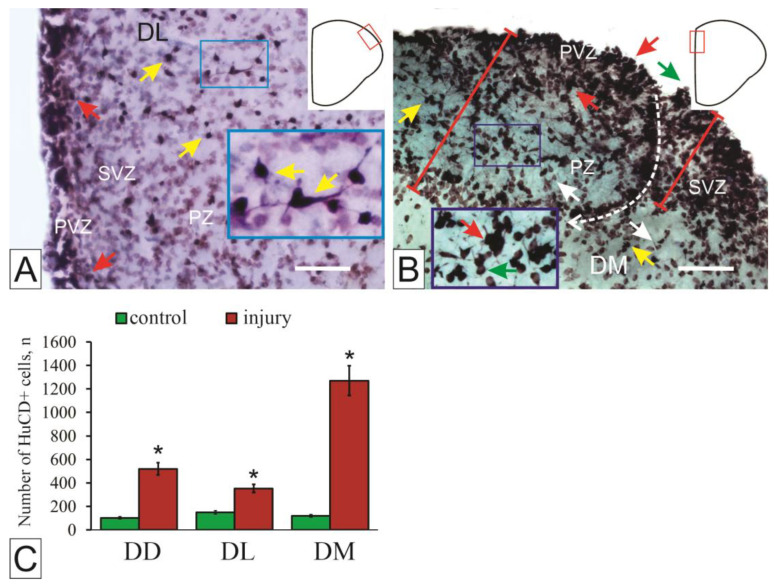
Expression of the neuronal protein HuCD (representative images) in the pallium of juvenile *O. masou* original image [50], (**A**) in intact animals, (**B**) after TBI; rectangles outline the insets in the figures, red arrows show intensely labeled neurons in the periventricular zone (PVZ) and subventricular zone (SVZ), yellow arrows show intensely labeled neurons in the parenchyma (PZ), green arrows show poorly labeled neurons in PVZ and SVZ, white arrows show poorly labeled neurons in PZ, white dotted line indicates the direction of cells migration, red segments show different thicknesses of the neurogenic layer. (**C**) the ratio of immunopositive cells in the control (intact animals) and after TBI (n = 5; * *p* < 0.05—compared with the control). Scale bar: 100 µm.

In the dorsal region of the intact telencephalon, juvenile HuCD+ cells were identified both in the outer proliferative zones of the telencephalon and in the deep layers (Figure 4A). The neuron-specific HuCD protein in the pallium of juvenile *O. masou* is detected in cells at various stages of neuronal differentiation. In the proliferative zones, HuCD labels neuronally determined progenitor cells, while in deeper layers it labels differentiated neurons of varying degrees of maturity (Figure 4A).

Three days after the injury, both intensively and moderately HuCD-immunopositive cells in deep layers were observed in the dorsal zone of the pallium, which significantly differed from the immunolabeling in the control (Figure 4B). A decrease in the intensity of HuCD immunolabeling in most definitive neurons, while maintaining intensive labeling in 25% of cells, is an expression of an adaptive response to mechanical injury in the telencephalon [50]. After the TBI, the proliferative zone underwent significant changes. Zones of induced neurogenesis were found, representing neurogenic niches of various morphology and, possibly, etiology (Figure 4B). As a result of the injury, a significant increase in the number of intensively labeled HuCD cells was found in the medial zone of the *O. masou* telencephalon (Figure 4C), located in the proliferative zone and formed streams of migrating neuronal specialization cells (Figure 4B). Such hyperproduction of neurons in the medial zone after injury generally corresponds to the increased proliferative potential of this zone as established by PCNA labeling [50].

Recent studies in *Mus musculus* have shown an increase in Pax6 expression in adult telencephalon, resulting in an increased number of TH-positive neurons in the granular layer of OLB [83,84]. Thus, for further comparison of neurogenesis in OLB in *D. rerio* and mammals, the ratio between the number of new TH neurons and the expression of Pax6 genes in *D. rerio* was analyzed. Two Pax6 genes with overlapping expression in embryos were identified in *D. rerio* [87], but their expression pattern in adults differed somewhat from that in the embryonic brain. While Pax6b expression was insignificant in the telencephalon and was absent in OLB, Pax6a was found in nearby cells, as well as in numerous cells in OLB. None of the cells expressing Pax6a were PCNA-positive. Labeling with polyclonal mouse antibodies showed no labeled cells in the ventricular zone. Double-labeled BrdU/Pax6a cells and a small number of TH/Pax6a-positive cells were detected in the OLB. Thus, populations of Pax6a- and TH-positive cells partially overlap.

Progenitor cells from the periventricular zone of the telencephalon in adult *D. rerio* also contribute to the replenishment of the neural pool of the telencephalon. In the subpallium and medial pallium, after a few weeks, these neurons arrange in dorso-ventral lines adjacent to the ventricular zone. Some neurochemical markers (GAD67, TH) and transcription factors (Pax6a) mark neurogenic areas of the brain [13]. The labeled cell columns are located side by side; these cells do not migrate further to the brain, but remain in the telencephalon and differentiate into adult neurons of the corresponding neurochemical specialization. Thus, telencephalon neurons and OLB neurons belong to the same subtype and share the same molecular markers.

Comparison of neurons from the dorsal subpallium and medial pallium, in particular, BrdU labeling and expression of the above markers showed that most of these neurons express GAD67. Pax6a or TH-positive neurons were also detected. However, TH-positive neurons do not express Pax6a or GAD67, unlike OLB neurons. Thus, the periventricular zone of the subpallium and ventral pallium gives rise to GABA or TH-positive telencephalic neurons.

## 8. Features of the Progenitor Cells of the Forebrain Ventricles

NSCs in the brain of adult mammals belong to a population of slowly proliferating cells and possess the properties of astrocytic glia [88,89]. To test whether similar properties are observed in the telencephalon of adult fish, the expression of brain lipid-binding protein (BLBP), expressed by mammalian and avian glial cells, was studied [90].

Of the two BLBP genes in *D. rerio*, fabp7a and fabp7b [91], fabp7a is expressed in the periventricular zone of the telencephalon and is functionally similar to the genes sox2, sox9a, sox10 and other markers of mouse and fish progenitor cells [92,93]. Fabp7a-positive cells were observed along the surface of the ventricle, spreading radial processes along the meninges. Two hours after BrdU labeling, 42% of the labeled progenitor cells also became fabp7a-positive. However, it is important that the percentage of fabp7a-positive and BrdU-labeled cells decreased over time, as BrdU-labeled cells migrate from the ventricular zone due to the acquisition of neuronal differentiation. Thus, progenitor cells from the periventricular zone of the *D. rerio* telencephalon have similar characteristics to mammalian NSCs, particularly in the expression of similar cellular and molecular glial factors.

**Figure 5:**
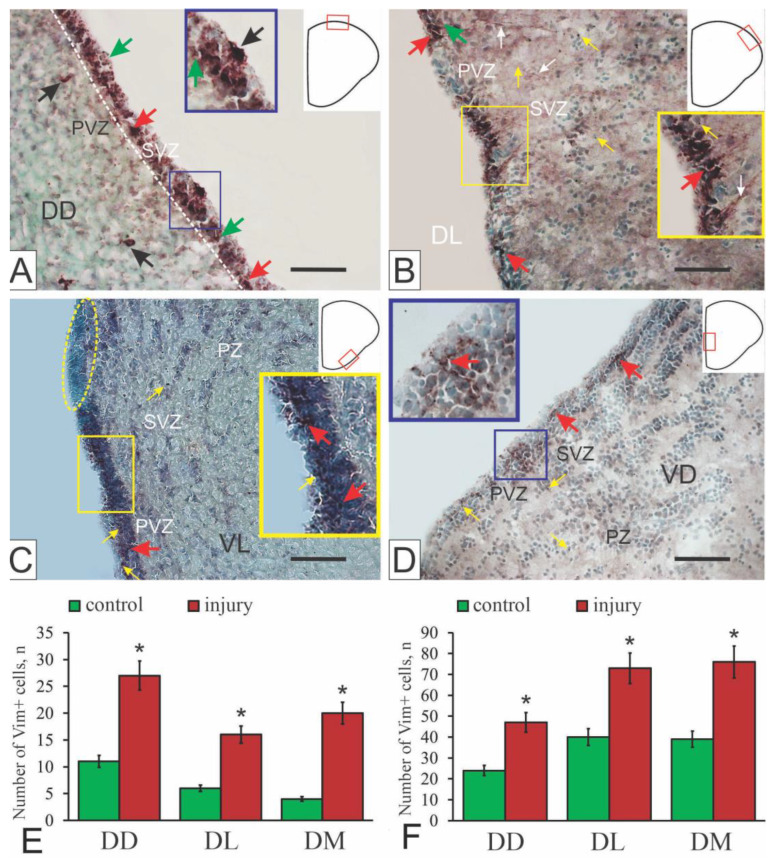
Representative distribution of vimentin in the pallial (**A**,**B**,**E**) and subpallial (**C**,**D**,**F**) proliferative zones of the telencephalon of juvenile *O. masou* original image [15,50]. (**A**,**B**)—in intact animals; (**C**,**D**)—after TBI; (**A**)—dorsal (DD), (**B**)—lateral (DL) zones of the pallium; rectangles outline the insets in the figures, the perventricular zone (PVZ), subventricular zone (SVZ), parenchyma (PP), red arrows show intensely labeled cells, black—intensely labeled elongated cells, green arrows—moderately labeled, yellow are immunolabeled extracellular granules, white are radial glial fibers, and the dotted line indicates PVZ. (**C**,**D**)—lateral (LV) and medial (VM) zones of the subpallium; rectangles outline the insets in the figures, yellow dotted oval outlines the immunonegativee cells, the rest of the designations as on (**A**,**B**). (**E**,**F**)—the ratio of immunopositive cells in the control (intact animal) and after TBI (n = 5; * *p* < 0.05—compared with the control). Scale bar: 100 µm.

IHC reaction products after vimentin labeling in the pallium of an intact *O. masou* were detected in cells of the periventricular area in the form of small granules located in various parts of the somas of immunopositive cells (Figure 5A). Individual intensively labeled cells, as a rule, formed small dense clusters or were arranged in a multi-row layer in the PVZ. Individual elongated cells were labelled in the PVZ (Figure 5A). Vimentin-positive cells of the glial phenotype are detected in the pallium of *D. rerio* [94]. However, in the pallium of *O. masou*, vimentin-positive cells corresponded to the glial progenitors of the adult type of aNSPCs (Figure 5A).

In the subpallial region of intact animals, vimentin immunolabeling was detected in the PVZ (Figure 5C). Intensely labeled intracellular granule-like inclusions of vimentin formed extended regions alternating with zones of immunonegativity (Figure 5C). Along with the group distribution patterns of vimentin, individual granules were labeled in the PVZ, and single Vim+ aNSPCs and granules were identified in the SVZ [16]. More extensive zones containing Vim+ aNSPCs and granules of parenchymal localization, located as part of local clusters of immunonegative cells, were identified in the PZ (Figure 5C).

After the TBI, vimentin expression in the aNSPCs of the PVZ increased (Figure 5B,E), which corresponds to the data on *D. rerio* [95]. After TBI, vimentin labeling was characterized by an increase in the number of immunopositive elements, including cells and granules, compared with intact vimentin expression (Figure 5B,D). An increase in the number of vimentin+ cells is typical for PVZ; clusters of vimentin+ cells without processes appeared, and patterns of vimentin+ clusters of RG emerged (Figure 5B). We believe that as a result of the TBI, an additional pool of vimentin+ aNSPCs and their descendants was activated in the *O. masou* pallium, aimed at eliminating the effects of trauma (Figure 5E,F).

Recent studies have shown that some molecular transcription factors, such as emx3 [96], dlx2a [97], Olig2 [98], Pax2 [99], ash1a [100], and neurog1 [101], are expressed by BrdU-positive progenitor cells in the telencephalon of adult *D. rerio*. The expression sites of these markers largely overlap and cover the entire periventricular zone in the subpallium and medial pallium. However, the dorsal and lateral inverted zones of the pallium express Pax6b and ash1a but do not express emx3, dlx2, olig2, and neurog1. These data indicate that progenitor cells located along the periventricular zone of the subpallium and medial pallium in adult fish express a combination of transcription factors different from that in embryonic neuronal cells. Thus, the combination of factors expressed by progenitor cells in *D. rerio* in the mature telencephalon is not equivalent to that in embryogenesis, and it is likely that the spatiotemporal pattern of these factors in the adult brain is created de novo.

## 9. Features of the Vimentin and Glial Fibrillary Acidic Protein Distribution in the Brain of Fish

Studies conducted on teleost fish revealed the presence of the intermediate filament protein vimentin (Vim) and glial fibrillary acidic protein (GFAP) in glial cells of the spinal cord and other brain regions [102]. In studies on the grey mullet *Chelon Labrosus*, the features of Vim and GFAP labeling in the brains of larvae, juveniles, and adults were studied. Different levels of expression of these proteins were observed throughout life. Thus, according to some data [102], the level of vimentin decreases with age, while the expression of GFAP increases. At earlier stages of fish development, labeling was usually expressed in cell soma, astrocyte pedicels, and tanycytes, whereas expression in RG cells was determined later. In large larvae, similar expression patterns of vimentin and GFAP suggested that some of the glial cells contained both proteins. Immunopositive for both proteins, subventricular cells were observed mainly in the optic tectum [103]. Immunopositive cells with astrocyte morphology were also found in the chiasm of adult grey mullet. Perivascular processes of glial cells had varying degrees of Vim and GFAP labeling at different stages of development. Some circumventricular organs (the vascular organ of the hypothalamus, *saccus vasculosis*, and posterior bay of the hypothalamus) were surrounded by highly specialized vimentin and/or GFAP-expressing glial cells. Glial cells of the median septum in some areas of the brain were also Vim+ and/or GFAP+. In the adult grey mullet brain, tanycytes retain vimentin expression in several brain regions. As in other vertebrates, regions with vimentin-immunopositive cells may correspond to areas with high plasticity and regenerative potential in the adult brain.

**Figure 6:**
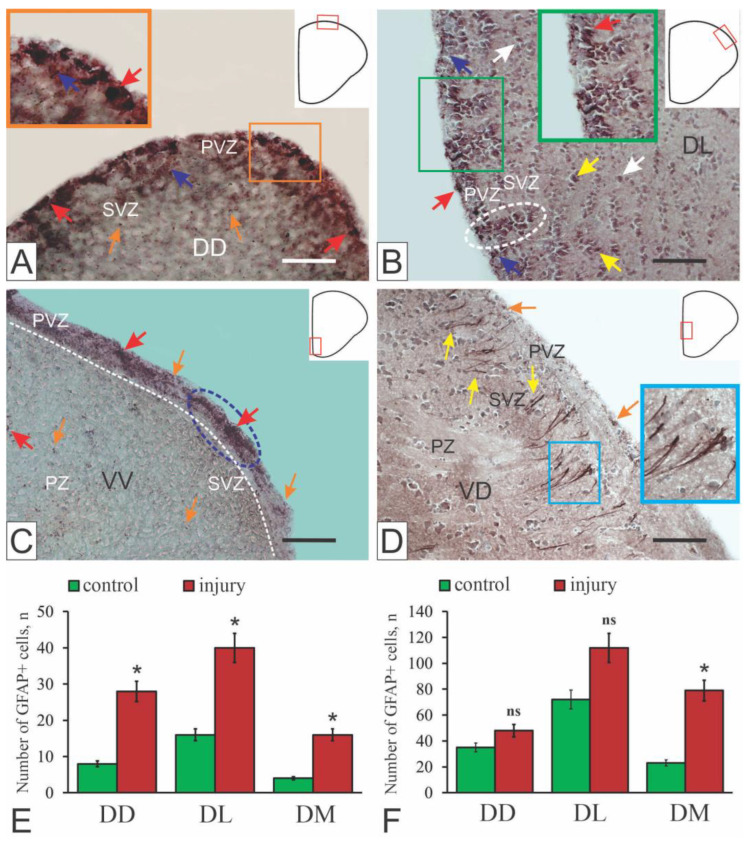
Representative distribution of GFAP in the pallial (**A**,**B**,**E**) and subpallial (**C**,**D**,**F**) proliferative zones of the telencephalon of juvenile *O. masou* original image [15,50]. (**A**,**B**)—in intact animals; (**C**,**D**)—after TBI; (**A**)—dorsal (DD), (**B**)—lateral (DL) zone of the pallium; rectangles outline the insets in the figures, red arrows show intensely labeled cells in the periventricular (PVZ) and subventricular (SVZ), yellow arrows show intensely labeled cells in the parenchyma (PZ), blue shows weakly labeled cells in PVZ and SVZ, white—weakly labeled in PZ, orange—labeled granules in PZ. (**B**)—ventral (VV), (**D**)—dorsal (DV) zones of the subpallium; The white dotted line indicates the PVZ, the blue dotted oval indicates constitutive clusters in the PVZ, the red arrows indicate intensively labeled cells, the yellow ones indicate radial glial fibers, and the orange ones indicate labeled granules. (**E**,**F**)—the ratio of immunopositive cells in the control (intact animals) and after TBI, ns—non significant (n = 5; * *p* < 0.05—compared with the control). Scale bar: 100 µm.

In studies of the telencephalon of intact juvenile *O. masou*, cells with a tanycyte-like morphology and/or typical process astrocytes were not detected in the proliferative zones (Figure 6A,C). However, single intensely GFAP-labeled cells were identified without signs of differentiation (Figure 6A,C).

After TBI, clusters of vimentin-positive cells, similar to GFAP-positive labeling, appeared (Figure 5B), along with numerous less intensely vimentin-labeled cells and single labeled cells in the brain parenchyma. Extended regions containing intensively labeled cells and radial glia were also observed [16,51]. As in the case of GFAP, vimentin labeling was characterized by the least evident structural changes and quantitative changes in the number of immunopositive cells (Figure 5B,E and Figure 6B,E).

As a result of the injury, structural rearrangements were found in the PVZ and SVZ, leading to the formation of reactive structural complexes, including a heterogeneous population of GFAP+ cells and radial glia (Figure 6B,E). The visible topological nature of such complexes, the differentiated organization of various cell types in them, and the high hierarchy in the ratio of individual elements suggest that these complexes correspond to reactive neurogenic niches containing glial precursors (aNSPCs) specific to adult neurogenesis, which are reactivated as a result of the traumatic injury [51].

The pattern of GFAP immunolabeling in juvenile *O. masou* after injury was significantly different from that of GFAP in intact animals (Figure 6A,B). Instead of single GFAP+ cells (Figure 6A,D), heterogeneous cell clusters, additional radial glial fibers [16], and single small intensely GFAP-labeled cells in the parenchyma were observed (Figure 6B,D). The total number of immunolabeled cells in all areas increased (Figure 6B,E). All these GFAP+ elements appeared de novo as a result of activation of glial-type resident aNSPCs and their subsequent slow proliferation in response to injury [16,51]. GFAP-immunopositive structures in the pallium and subpallium of juveniles are reactive neurogenic niches containing glial-type aNSPCs that arise in response to injury [16,51].

In studies on *D. rerio*, radial glial aNSPCs and intermediate precursors in the pallium were identified [103]. The source of aNSPCs is embryonic radial glia [104], which produce intermediate progenitor cells [105], characterized by high heterogeneity in terms of active cycling or dormancy and by expressed molecular markers.

The following types of glial cells are distinguished in the central nervous system: astroglia, oligodendroglia, and microglia. In the central nervous system of adult mammals, astroglia include protoplasmic and fibrous astrocytes located in gray and white matter, respectively; radial glial cells; and ependymal cells [106]. Studies of the ultrastructure of these cells revealed the presence of intermediate filaments [107]. Biochemical analysis and immunocytochemical studies have shown the presence of GFAP in the intermediate filaments of astrocytes [108]. Studies have shown similarities between the GFAP of bony fish and that of mammals [109]. Another intermediate filament protein, vimentin, was also found in astroglial cells [110]. It is expressed in large quantities of immature astrocytes. Sequencing of bony fish vimentin and analysis of its amino acid sequence showed a high degree of homology with the human protein [94]. Antibodies against these two proteins are currently used as markers of astroglia.

RG cells, whose processes extend from the subventricular region to the meninges, are abundant during mammalian brain development. These cells are considered an intermediate stage in the maturation of astrocytes. RG cells form a scaffold for neuron migration [111]. In most species, these cells express GFAP [57]. Recent data show that radial glial cells have diverse functions. There is evidence that they are progenitor cells of neurons and glia [88,112].

Astrocytes have been found in most vertebrates. In teleost fish, however, astroglia are mainly represented by ependymal tanycytes and RGs [113,114]. Astrocytes have been described in the brain of bony fish [113,115], and reticular astrocytes with epithelial cell characteristics are common in the optic nerve [116].

Most ependymal cells of adult bony fish are tanycytes [117], that is, they have long basal processes terminating on the outer surface of the brain [118]. Immunocytochemical studies of ependymal cells and RG of bony fish have demonstrated the presence of GFAP [119,120] and vimentin [94,121]. NADPH-diaphorase [122] has been used to describe the distribution of tanycytes in the adult brain of moonfish (*Lepomis* sp.). These glial populations have also been studied using S100 and calbindin in trout [114]. The early development of GFAP-immunopositive cells was studied on *D. rerio* embryos [57]. However, to date, there are no studies on changes in the pattern of GFAP- and Vim-immunopositive cells in the brains of bony fish.

## 10. The Pax Family of Genes, Their Involvement in Nervous System Development, and Their Potential Role in Neuroregeneration

The main feature of organogenesis is the spatiotemporal organization of cell migration, which is a necessary condition for the formation of various cell types. Pax genes encode a family of transcription factors that have long been recognized as essential participants in the embryonic development of the central nervous system, with evidence from various animal models illustrating phylogenetically conserved functions. Within the CNS, Pax genes play a crucial role in cellular and regional specification, proliferation, maintenance of progenitor cells, antiapoptosis, and neuronal differentiation.

The Pax gene family demonstrates dynamic spatiotemporal expression patterns and, together with other factors, participates in coordinating the regional development of the CNS, identifying neural subtypes and controlling their migration and differentiation [123].

Throughout life, numerous different progenitor cells expressing Pax family genes persist in the brain until environmental stimuli trigger their proliferation or differentiation processes [123]. The Pax gene family demonstrates dynamic spatiotemporal expression patterns and, together with other factors, participates in coordinating the regional development of the CNS, identifying neural subtypes and controlling their migration and differentiation [124]. Therefore, the Pax family is important for organization and migration of cells throughout all stages of CNS development and maturation.

The genes of the Pax family are expressed at the earliest stages of the development of the nervous system: gastrulation and formation of the neural plate [125]. Pax genes are key regulators of brain regionalization processes, controlling the synthesis of signaling molecules and thereby contributing to the formation of a specific cell phenotype [126]. Their differential expression depends on the spatial localization of expressing cells in signaling centers, and the levels of Pax expression determine the emergence of various cellular phenotypes. This mechanism is particularly important during embryonic development of the CNS.

Pax3 and Pax7 are expressed on the dorsal edges of the early neural plate preceding the closure of the neural tube, where they guide cells along the entire neural tube. Cells of the dorsal part of the neural tube form sensory neurons and interneurons, as well as neural crest cells [127]. Pax6 is expressed throughout the middle ventral region of the developing neural tube, generating motor neurons and interneurons [128]. Pax2 is expressed in the neural tube, at the intermediate dorsoventral border of the rhombencephalon and spinal cord [99], producing interneurons of the posterior rhombencephalon and spinal cord [129].

Studies of the Pax gene expression have demonstrated a highly specific anterior–posterior pattern during the regionalization of the developing CNS [130]. Initially, the differential expression of Pax6 and Pax2 divides the neural tube into three primary domains (prosencephalon, mesencephalon, and rhombencephalon) [130]. Subsequently, interactions among Pax genes, as well as between Pax genes and other genes, contribute to determining polarity, establishing boundaries, and specifying progenitor cells in the ventricular zones to form nuclei and related structures. The ability of Pax genes to mutually suppress the expression of genes of the alternative Pax group leads to zones of exclusivity for each Pax gene or group of Pax genes. Pax proteins can also interact with other transcription factors (for example, the opposite gradients of Pax6 and Emx2 or Pax6 and Dlx2 in the cerebral cortex, and Pax6 and Olig2 in the olfactory bulb/Pax6 with cVax and Tbx5 in the retina) to achieve the specification of cellular subtypes and the formation of boundaries [131].

The complex activity of Pax genes is clearly coordinated to achieve differentiated regulatory mechanisms at different times and in different locations. For example, the cooperative and excessive activity of Pax6 and Pax2 indicates retinal pigment epithelium at early stages of development [132], while they are mutually repressive at later stages [133]. Accordingly, the mutually coordinated repression between Pax6 and Pax2/5/8 regulates the development of spinal cord interneurons [134]. Thus, synchronous and highly coordinated Pax expression is crucial for determining the early processes of pattern formation in the development of the CNS. The critical importance of Pax genes is well illustrated in mutant animal models, where the absence of Pax transcription factors leads to disruption of histogenetic patterns and loss of cells and structures. Moreover, conditional mutant models (absence/decrease/overexpression) allowed us to understand the functions of Pax at different stages of development. Obviously, the analysis of such valuable animal models will provide a deeper understanding of the influence of Pax genes throughout different stages of development [135].

Pax genes promote extensive cell migration. For example, neural crest cells expressing Pax3 migrate intensively along the body, and Pax3 deficiency leads to a change in migration or a decrease in the number of cells in the target location [136]. Proper migration of some cell populations requires Pax6; its deficiency in mice can result in altered neuroblast migration in the developing cerebral cortex [137] and cerebellum [138]. However, a reduced number of cells in a certain area is not always caused by disruptions in cell migration, but may also be a consequence of insufficient expansion of the progenitor pool. The expression of Pax genes not only directs cells to new organs and tissues but is also necessary to maintain them in a progenitor state during migration.

Differences between autonomous and non-autonomous cellular interactions with Pax genes involved in regulating migration are achieved by transplantation of Pax-deficient cells in the body (under normal/abnormal conditions). The role of Pax in non-autonomous migration is clearly demonstrated by studies in which Pax6-deficient neural crest cells in rats fail to migrate properly to the eye and craniofacial region [139]. Transplantation of neural crest–derived brain cells from wild rats into a Pax6-deficient environment does not restore normal migration, indicating that the defect arises from a mis-specified migration pathway rather than a shortage of migrating cells [139]. This conclusion is supported by the observation that late-born Pax6-deficient progenitor cells of the cerebral cortex display similar migration behavior when transplanted.

Pax genes also contribute to migration processes in developing tissues, such as the direction of axons [140,141]. During cerebral cortex development, precursors from the subventricular zone migrate to their respective destinations, using PSA-NCAM and robo2 as guiding molecules. In Pax6 experimental mice, qualitative changes in the PSA-NCAM pathways in the intermediate zone were detected, manifested as delayed or suppressed robo2 expression and subsequent migration deficiency [141]. Moreover, Pax6-expressing cells of the *medulla oblongata* of the rat embryo are bound to TAG1 cell adhesion molecules and migrate along axons that express TAG 1. In this region, in Pax6-deficient rats, TAG 1 expression is suspended and subpopulations of these cells migrate incorrectly [138].

Although Pax2 regulation of N-CAM and N-cadherin cell adhesion molecules is more related to morphogenesis than to migration, studies of chick eye development clearly demonstrate that Pax2 knockdown leads to the absence of N-CAM and N-cadherin molecules, whereas Pax2 overexpression regulates these molecules [142].

This analysis of Pax migration capability indicates the great influence of Pax genes on the direction of cell migration, both in the embryonic and postnatal environment. This property can be used to guide cells to the desired destination or to block their migration in previously transplanted cells.

After primary regionalization of the CNS is complete, the areas expressing Pax in the brain become more limited. The main contribution of Pax genes is to maintain a complex balance between cell proliferation, preservation of the proliferative pool, and differentiation of progenitor cells.

For example, Pax6 controls the expansion of the proliferative pool in developing areas such as the ocular vesicle [143], cerebral cortex [135], and hippocampus [144]. Pax6 also promotes the proliferation of retinal stem cells and supports the proliferative pool throughout several stages of *Xenopus* retinogenesis [145]. A decrease in Pax6 levels leads to reduced proliferation and/or premature differentiation of neurogenic precursors during the formation of the eyes [143], cerebral cortex [135], spinal cord [146] and hippocampus [147]. These results show that Pax6 levels mediate critical spatiotemporal synchronization of progenitor cell proliferation and differentiation dynamics to ensure proper formation of CNS regions.

**Figure 7:**
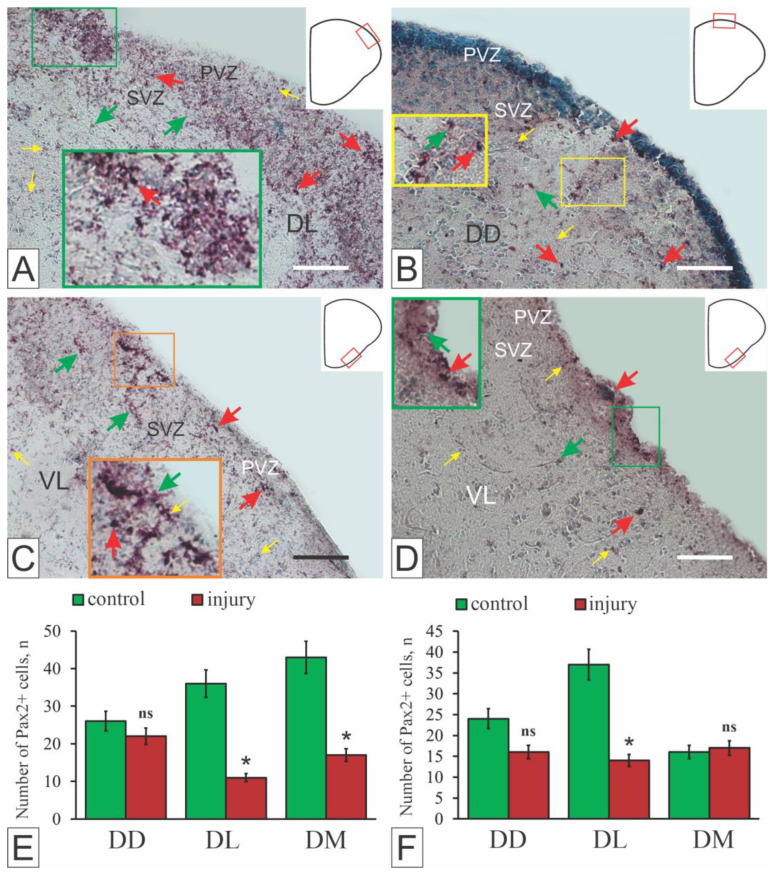
Representative Pax2 distributions in the pallial (**A**,**B**,**E**) and subpallial (**C**,**D**,**F**) proliferative zones of the telencephalon of juvenile *O. keta* original image [15]. (**A**,**B**)—in intact animals; (**C**,**D**)—after mechanical injury; (**A**)—lateral (DL), (**B**)—dorsal (DD) zone of the pallium, (**C**,**D**)—lateral (VL) zone of the subpallium; rectangles outline the insets in the figures, the perventricular zone (PVZ), the subventricular zone (SVZ), the parenchyma (PZ), red arrows show intensively marked cells, green—moderately labeled, yellow are immunolabeled extracellular granules. (**E**,**F**)—the ratio of immunopositive cells in the control (intact animals) and after injury ns—non significant (n = 5; * *p* < 0.05—compared with the control). Scale bar: 100 µm.

In the telencephalon of juvenile chum salmon *Oncorhynchus keta*, the protein product Pax2 exhibited both cytoplasmic and nuclear localization (Figure 7A–D), resulting in a heterogeneous population of Pax2+ cells and granules [15]. In intact animals, Pax2+ cells were detected as part of labeled cell clusters in the PVZ of the pallial and subpallial regions, in less dense subventricular groups, and as single superficially localized cells (Figure 7A,C). However, in most areas of the pallial region, the Pax2 protein product was labeled in the cell nuclei. The regions containing the Pax2-labeled nuclei had a spatially extended structure, forming morphogenetic fields with varying intensity of immunolabeling, and the pattern of cell distribution was diffuse (Figure 7A–D).

A comparative analysis of Pax2 expression in the telencephalon of intact juvenile *O. keta* showed constitutive Pax2 expression patterns in neurogenic regions and non-neurogenic parenchymal zones of the pallium and subpallium [15]. After mechanical injury, the nature of Pax2 expression changed, and the amount of Pax2+ cells decreased (*p* < 0.05) in the lateral (Dl), medial (Dm) zones of the pallium (Figure 7E) and the lateral zone of the subpallium (Vl, Figure 7F) compared with the control. It is possible that the decrease in Pax2 expression was due to the inhibitory effect of the transcription factor Pax6, the expression of which in the brain of juveniles of another representative of salmonids, trout, increases after injury [148].

In juvenile chum salmon, Pax2 was found to label a population of neuroepithelial cells (NECs) in various regions of the telencephalon. Pax2 was expressed both in the cytoplasm and in the nuclei of these cells [15]. The Pax2 protein product labels a limited population of NECs and cell nuclei of unknown functional specialization involved in the constitutive neurogenesis. In juveniles of *O. masou*, Pax6 immunolocalization reveals the neuromeric organization of the brain at different stages of postnatal ontogenesis [149,150]. Taking into account the data on the involvement of Pax family genes in constructing the spatial structure of the CNS in embryogenesis and postnatal development [151], we believe that the expression of Pax2 in cell nuclei in the deep parenchymal layers reflects brain patterning in growing juvenile chum salmon. This assumption is consistent with data on the involvement of Pax2 in the process of CNS regionalization during the embryonic and postembryonic periods of vertebrate development [151].

Further complication of the developmental process is ensured by the coordinated expression of many Pax genes, which may be required for proper development and final determination of cells. For example, Pax6 and Pax2 are co-expressed during the formation of CNS boundaries. Another classic example is their consistent expression during eye development.

At an early stage of ocular bladder development, the coordinated and excessive activity of Pax6 and Pax2 determines the retinal pigment epithelium [132]. The divergent nature of expression at slightly later stages defines the boundaries between the retina (Pax6-positive) and the optic nerve (Pax2-positive) [133]. Experimental suppression of Pax2 in the optic nerve of a mouse embryo explains the reasons for the regulation of ectopic Pax6 expression and ectopic neuronal differentiation [152]. With the development of the spinal cord, Pax2 provides Lnx1/Lnx5 and Pax5/8 expression of dorsal horn interneurons for proper neuronal specification [134]. In the ventral dorsal horn, Pax2 expression is triggered when cells become postmitotic and later migrate to the mantle zone. However, before the appearance of postmitotic neurons, Pax6 is required for the initial specification of neuronal progenitor cells and thus regulates the expression of Pax2 and other neuronal genes. Therefore, the coordinated expression of Pax6 and Pax2 is necessary for the correct specification of identical ventral interneurons.

Taken together, the Pax genes, providing neural cellular phenotypes, are involved in determining the time of exit from the cell cycle and, thus, regulate the differentiation of the corresponding cell types under suitable spatiotemporal conditions. In some cases, Pax genes cooperate with other members of the Pax family and/or other cofactors. The main task in deciphering the functions of Pax and their restorative properties is to identify the mechanisms underlying the transition of Pax functions from promoting proliferation to supporting differentiation. Pax genes are a critical factor influencing all stages of development, from the beginning of progenitor expansion to ensuring and supporting proper neuronal differentiation. Together, these results also reveal an important property of Pax genes: their ability to act as a powerful spatiotemporal programming switch sensitive to environmental conditions. This forces us to consider them as an important factor ensuring the restructuring of the CNS. Insights from these studies will significantly aid in deciphering the genetic/epigenetic environmental factors involved in the suppression of Pax functions at various temporal and spatial levels of development.

One of the important aspects of neuroscience research is identifying factors that influence a cell’s ability to survive neurotrauma or neurodegeneration and subsequent inflammatory processes. Although information on the role of Pax genes in this context is limited, several studies have demonstrated that Pax-expressing cells can respond to neurotrauma and contribute to creating a favorable environment following a stroke [153].

Studies have demonstrated the ability of newborn Pax6-expressing neural progenitor cells to survive in the long term both in the SGZ of the hippocampal *dentate gyrus* and in the SVZ [154]. Similarly, Pax-expressing cells survive damage in various tissues: Pax6- and Pax7-expressing cells remain in the damaged spinal cord of adult rats [155], Pax6 expression increases in the postnatal olfactory epithelium [153] and resumes in retinal cells, including Müller cells [156]. Collectively, these results indicate that Pax-expressing cells are capable of long-term survival and can modulate the microenvironment in the brain following injury.

Thus, Pax genes are involved in almost all aspects of CNS development, from early to mature stages. While their function in adult differentiated cells remains largely unknown, there is ample evidence of the important role of Pax genes in controlling and ensuring consistency in many aspects of neuronal maturation.

First, Pax genes direct organogenesis, providing a sufficient number of progenitor cells for developing tissues. This property can be used for stem cell therapy to support initial cell growth (if the culture develops in vitro). Second, Pax genes maintain cells in an undifferentiated state until differentiation begins, providing spatiotemporal control over the generation of mature cell types within dynamic developmental niches. This characterizes Pax genes as multipotent switches that guide cells along differentiation paths determined by their spatial positional information. Similarly, Pax-expressing cells respond to spatiotemporal cues from the external environment, creating conditions that support pre-established Pax functions and enabling the integration of signals from both the external and internal cellular environment.

## 11. Functions of Glutamine Synthetase in the Central Nervous System

GS is a multifunctional enzyme involved in amino acid balance, nucleotide biosynthesis, neurotransmitter metabolism, and ammonia detoxication [157]. The glutamine synthetase gene (glul) is considered one of the oldest known functional genes [158], with expression observed in all studied species and tissues. GS activity in the brain of vertebrates, including fish, is usually high [157,159], as this enzyme plays a major role in the metabolic regulation of the neurotransmitter glutamate [9], as well as the detoxification of ammonia [160].

The brain is particularly vulnerable to ammonia toxicity for two reasons: it lacks a functional urea cycle, and ammonia can easily cross the blood–brain barrier. Accordingly, GS is mainly expressed in astrocytes (in vivo and in vitro) [161], which act as the main tool for removing ammonia [162]. The role of astrocytes in ammonia detoxification is further emphasized by their anatomical proximity to the blood–brain barrier [162].

For a long time, GS was considered a marker of astroglial cells, but now it has been shown that GS expression is not limited to astrocytes only. GS is also present in the Müller glia of the retina [163], in the cells of the RG and ependymoglial cells that line the ventricles in lower vertebrates [161]. In addition, GS can be detected in oligodendrocytes both in vivo and in vitro [164]. In fish, GS-immunopositive cells are located in the ependymal layer [165]. The expression of GS was not observed in all areas of the cell, indicating the regional specialization of the fish RG. Studies on trout have shown that the cerebral activity of GS in mammals is an order of magnitude lower than in fish [166].

It is generally assumed that GS activity is regulated during development [167]. In rats, the expression level of GS increases during development [168], which is associated with astrocyte differentiation [169]. In addition, one of the key roles of astrocytes is to protect neurons from glutamate toxicity. GS is an enzyme of crucial neurochemical importance because it converts toxic L-glutamate into neutral L-glutamine. L-glutamate is incorporated into vesicles in the synaptic cleft and released upon stimulation. After its function is completed, glutamate is absorbed by astrocytes, where it is converted to L-glutamine and recycled back to the vesicles of the neuron, allowing reconversion to glutamate [170]. In this cycle, glutamate is not metabolized, but only recirculated, in order to avoid the toxic effects observed in many diseases [171] associated with hyperstimulation of neurons due to excessive accumulation of amino acids. GS in the brain also plays a major role in the glutamine-glutamate-GABA cycle, maintaining a balance of excitatory and inhibitory synaptic transmission through synthesis, cellular release, and extracellular uptake of glutamate and GABA [172].

**Figure 8:**
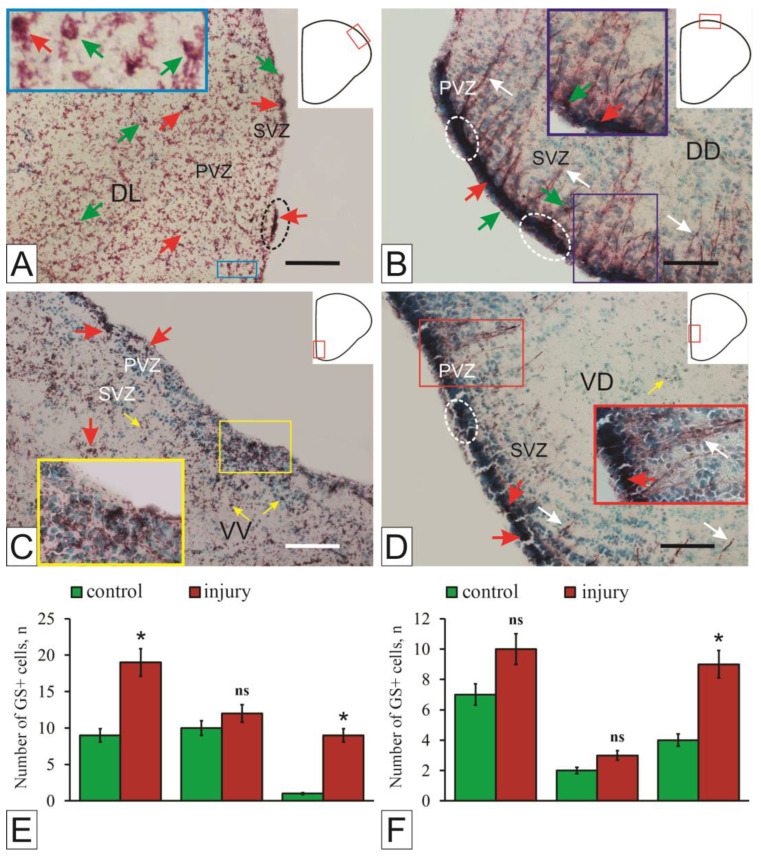
Representative distribution of GS in the pallial (**A**,**B**,**E**) and subpallial (**C**,**D**,**F**) proliferative zones of the telencephalon of juvenile *O. keta* original image [15]. (**A**,**B**)—in intact animal; (**C**,**D**)—after mechanical injury; (**A**)—lateral (DL), (**B**)—dorsal (DD) zones of the pallium; rectangles outline the insets in the figures, the perventricular zone (PVZ), subventricular zone (SVZ), parenchyma (PZ), red arrows show intensely labeled cells, green—moderately labeled, white—radial glial fibers. The black dotted line indicates the accumulation of immunopositive cells in the PVZ, and the white line indicates reactive neurogenic niches. (**B**)—ventral (BB), (**D**)—dorsal (DV) zones of the subpallium; yellow arrows show immunolabeled extracellular granules. (**E**,**F**)—the ratio of immunopositive cells in the control (intact animal) and after injury ns—non significant (n = 5; * *p* < 0.05—compared with the control). Scale bar: 100 µm.

In studies on juvenile chum salmon, GS expression was detected in the pallial and subpallial regions of the telencephalon [15]. In the intact brain, GS labeled a heterogeneous population of neuronal progenitor cells localized in the PVZ, as well as a limited number of aNSPCs in the parenchyma (Figure 8A,B). GS-expressing cells also exhibited astrocytic morphology (Figure 8A) and were found either individually or in clusters (Figure 8A,B).

Significant changes in the localization of GS in the telencephalon of juvenile chum salmon 3 days after the traumatic injury were revealed (Figure 8B,D). An additional type of GS+ neuronal precursors with a RG phenotype, absent in intact animals, appeared (Figure 8B,D). TBI to the telencephalon led to a significant increase in the number of GS+ cells in PVZ (Figure 8 E,F). Under injury conditions, the heterogeneous population of GS+ cells included two types of neuronal precursors [15]. NECs, previously present in the constitutive neurogenic niche were involved in the process of reactivation and formation of the reactive neurogenic niche (RNN). Another type of neuronal progenitor, absent in intact animals, was a heterogeneous population of radial glia detected in the RNN in various combinations [15]. The distribution density of reactive RG cells in the pallial and subpallal regions of the telencephalon increased significantly after injury, but significant differences were found in the dorsal and medial regions of the pallium (Figure 8B,E) and the ventral region of the subpallium (Figure 8D,F).

The results of studies on juvenile chum salmon corresponded to data on other fish species: *Apteronotus* [17] and *Danio rerio* [7]. Unlike in mammals, after brain damage in fish, GS activity increases, which ultimately helps to remove toxic glutamate from the intercellular space and create a favorable environment for reparative processes [9].

Since GS activity affects not only the brain’s ability to remove ammonia but also the glutamate cycle, variations in GS immunoreactivity reflect changes in astrocyte function and can influence neuron function. Glial cells expressing GS can also have an effect in pathological conditions of the brain. Changes in GS levels have been described depending on the type of injury. For example, GS activity decreases in the cerebral cortex affected by Alzheimer’s disease [173], during aging [174], and under glucose deficiency [175]. However, GS expression increases under ischemia and hypoxia [176].

The role of GS in the brain has been highlighted in the study of neurodegenerative disorders [177]. Because of its particular sensitivity to oxidizing agents [178], GS activity has been recognized as an indicator of the harmful effects of reactive oxygen species, which lead to brain damage. In mammals, microglia, similarly to astrocytes, express the glutamate removal system, also known as cellular glutamate transporter 1 (GLT-1) and GS, both under physiologically normal and pathological conditions [179]. The presence of this system places microglia on a par with astrocytes in protecting neurons from toxic glutamate. Moreover, the absorption of glutamate from extracellular spaces can promote the production of glutathione by co-expression of GLT-1 [180]. In microglial cells, the conversion of glutamate to glutamine can modulate the cellular response to an inflammatory stimulus [180]. Microglia can metabolically control their own response to a pro-inflammatory stimulus by converting glutamate to glutamine [180].

These data confirm that GS sensitivity to redox balance may represent a strategy by which various mechanisms related to the inflammatory response are modulated [180]. In addition, this proves that the beneficial role of GS in the brain is not limited to its ability to remove ammonia and glutamate, but also contributes to the maintenance of microglia in an immunosuppressive state.

Considering the important role of GS in the CNS during injury, an increase in its level in the cerebellum of fish after mechanical injury is particularly notable [181]. This likely reflects differences in the extent of neuronal death across taxa. It has been experimentally proven that increasing the level of GS in mammalian retinal glial cells, either through endogenous induction of a gene or exogenous administration of a purified enzyme, can protect cells from neural degeneration [182].

## 12. Cystathionine-β-Synthase and Hydrogen Sulfide in the Central Nervous System

Currently, there is increasing evidence that the signaling pathways regulating homeostasis in vertebrates are numerous and diverse. Hydrogen sulfide (H_2_S) plays a significant role in this context, and relatively high concentrations of this gas have been found in the brain, suggesting its physiological importance [183,184]. Subsequently, the most important biological effects of H_2_S were discovered, including regulation of blood pressure, release of insulin, vasorelaxation, modulation of neural activity, cytoprotection [185]. These properties allowed it to be attributed to the group of gas transmitters, which also include nitric oxide (II) (NO) and carbon monoxide (CO) [185,186].

CBS is the primary source of H_2_S in the CNS. The substrate for the synthesis of endogenous H_2_S is the sulfur-containing amino acid L-cysteine, which is obtained from food or synthesized from L-methionine by the so-called transsulfuration, forming homocysteine as an intermediate. The main mechanism of H_2_S production involving CBS is associated with the condensation of homocysteine and cysteine, which leads to the formation of cystathionine, with the concomitant release of H_2_S.

Brain homogenates have been shown to produce H_2_S in vitro [183]. High levels of CBS expression were found in the hippocampus of rats and the cerebellum of fish. Elevated expression during embryonic and early postnatal development is probably necessary for the maturation and growth of neural networks.

H_2_S is able to regulate the activity of GABA receptors located both pre- and postsynaptically [187]. Stimulation of postsynaptic GABA receptors induces long-term depression of postsynaptic transmission. This is due to an increase in K+ levels and is an essential factor in fine-tuning inhibitory neurotransmission. In neurons of the dorsal suture nucleus and in the hippocampus, H_2_S participates in the induction of hyperpolarization by increasing the influx of K+ through ATP-dependent potassium channels. H_2_S is also involved in the regulation of blood pressure by influencing KATP channels in hypothalamic neurons [188].

In addition to participating in neuromodulation processes, H_2_S is involved in protecting neurons from oxidative stress. It is known that reduced glutathione acts as one of the main antioxidant protectors of the brain. Its protective function involves the efficient capture of free radicals and other reactive groups, as well as the removal of hydrogen peroxide and lipid peroxides, thereby preventing the oxidation of other biomolecules [189].

H_2_S plays an important neuromodulatory role in glial cells. Astrocytes are necessary for maintaining the normal physiological properties of neurons, since these glial elements are able to regulate acid-base homeostasis and absorb various neurotransmitters, including L-glutamate [190]. Unlike neurons, which transmit signals mainly by generating action potentials, astrocytes and other glial cells “communicate” with each other through calcium signaling. The modulation of neuronal and vascular states is largely based on this phenomenon [190].

Exogenous H_2_S has been shown to cause calcium waves in the primary culture of astrocytes and in surviving sections of the hippocampus. Comparative analysis showed that in the CNS, maximum expression was observed in the ventricular regions with proliferating cells, in the striatum, in the IV ventricle, in the *medulla oblongata*, whereas weaker signals were detected in the midbrain, neocortex, and spinal cord. Immunohistochemically, it has been shown that CBS is localized not only in the neuron body but also in dendrites, axons, and synaptic terminals [191]. It was found that in masu salmon *Oncorhynchus masou*, CBS marks neurons of the reticular formation, vessels, neurons of the ventral spinal column and climbing fibers in the cerebellum [192,193].

**Figure 9:**
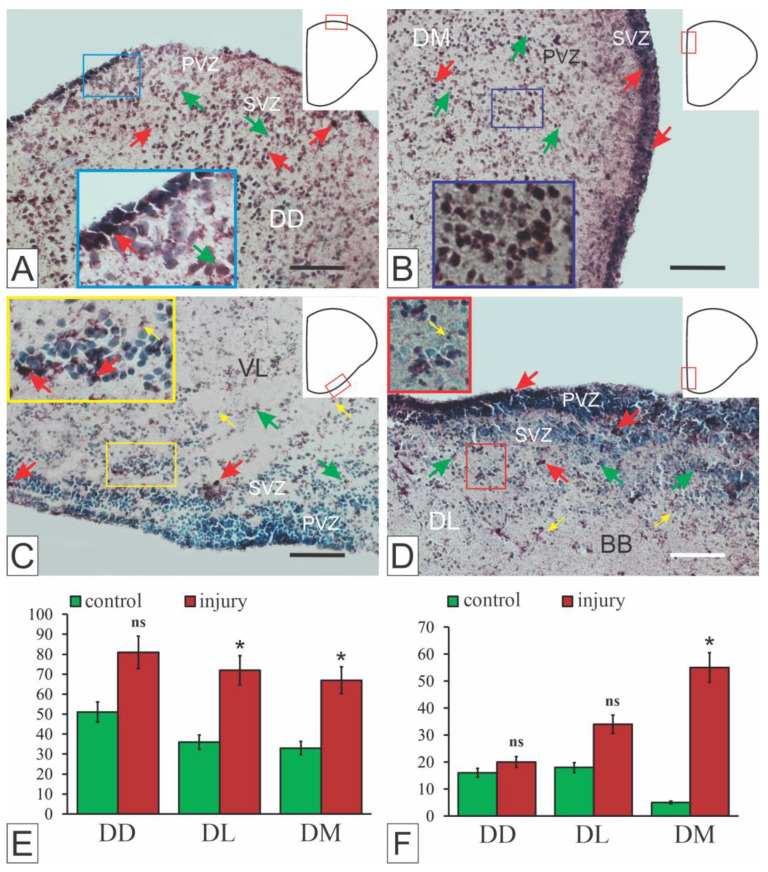
Representative CBS distribution in the pallial (**A**,**B**,**E**) and subpallial (**C**,**D**,**F**) proliferative zones of the telencephalon of juvenile *O. keta* original image [15]. (**A**,**B**)—in intact animals; (**C**,**D**)—after mechanical injury; (**A**)—dorsal (DD), (**B**)—medial (DM) zones of the pallium, (**C**)—lateral (VL), (**D**)—ventral (VV) zones of the subpallium; rectangles outline the insets in the figures, the perventricular zone (PVZ), subventricular zone (SVZ), parenchyma (PZ), the red arrows show intensively labeled cells, green—moderately labeled, yellow—immunolabeled extracellular granules. (**E**,**F**)—the ratio of immunopositive cells in the control (intact animals) and after injury ns—non significant (n = 5; * *p* < 0.05—compared with the control). Scale bar: 100 µm.

In the pallium of intact chum salmon *O. keta*, CBS labeling was detected in all zones [15]. The distribution pattern of CBS+ cells in the pallium was represented by a multi-row layer of intensely labeled cells (Figure 9A). Small clusters of CBS+ cells were recorded in DM as part of PVZ and SVZ; the number of labeled cells of parenchymal localization was located discretely or in small clusters (Figure 9A). The features of cell labeling in all areas of the dorsal region made it possible to identify both single immunolabeled cells and small CBS+ clusters in the PVZ (Figure 9A, inset). The maximum distribution density of intensely and moderately labeled cells was observed in the DD, whereas in the DL and DM the indicators were slightly lower (Figure 9E).

In the subpallial region of intact juvenile chum salmon, CBS immunopositivity was also detected in all zones (Figure 9B). CBS-negative cells predominated in the SVZ, among which small clusters of small elongated basophilic cells were observed (Figure 9D). In the deeper proliferative zone (PZ), clusters of moderately and intensely labeled CBS+ cells, either as small groups or single cells, were present alongside CBS-negative small basophilic cells (Figure 9B).

After telencephalon TBI, the number of CBS-labeled cells in the PVZ of the subpallium increased significantly compared with control animals (Figure 9D,F). The morphological characteristics of the cells changed; in general, smaller cells prevailed in all areas after injury. In the subpallial region, similar rearrangements occurred in the dorsal (VD) and ventral (Vv) nuclei (Figure 9C,D). The number of individual and paired CBS+ cells, as well as their small clusters, increased in the subpallium (Figure 9D).

Currently, the involvement of H_2_S in the process of ischemic brain injury, TBI, and the involvement of this gas transmitter in the control of oxidative stress and an increase in reactive oxygen species in H_2_S-dependent signaling are being investigated [194]. However, information on intercellular interaction and the involvement of H_2_S in regenerative processes, in particular adult neurogenesis and traumatic brain injury, is still limited.

Studies have shown that in the dorsal and ventral parts of the telencephalon of juvenile masu salmon and chum salmon, CBS marks a heterogeneous population of periventrically and parenchymally located cells [15,192]. Despite regional differences, common features of CBS localization were identified: extended distribution zones of intensively labeled cells alternated with areas devoid of immunopositivity, which corresponds to the patterns of GS distribution in neuroepithelial cells in intact telencephalon (Figure 9B,E).

The results of CBS immunolabeling in intact chum salmon juveniles largely correspond to the pattern of HuCD distribution in juvenile *O. masou*, suggesting the possible involvement of H_2_S in the regulation of adult neurogenesis [15,192]. This assumption is further supported by the presence of numerous HuCD+ neurons at various stages of differentiation in the parenchyma of the dorsal region, surrounded by CBS-positive neurons localized in the same areas.

In the absence of a differentiated cellular structure in the telencephalon, H_2_S-producing cells in the brain parenchyma secrete H_2_S into the intercellular space, where young neurons differentiate, thereby creating a special microenvironment conducive to neuronal differentiation. H_2_S secreted by cells can be considered a factor involved in constitutive neurogenesis. According to the data, a high level of CBS is necessary for the early maturation and growth of neural networks, which also confirms the role of hydrogen sulfide in neuronal differentiation [193].

The presence of the H_2_S-producing enzyme in brain cells and probably in granules is associated with neurochemical signaling processes, in particular with the activation of NMDA receptors [194,195]. Activation of neurons in the brain leads to the release of neurotransmitters, including glutamate, which activates NMDA receptors and, in turn, increases astrocytic intracellular calcium and long-term potentiation [196]. The presence of two levels of CBS activity in telencephalon cells and granules indicates mediator-modulatory intercellular interactions, consistent with previously obtained data in fish [197].

Microglial cells are known to be activated by many external influences [198]. Changes in the state of microglia are considered significant pathogenetic factors in the development of Alzheimer’s disease [199] and Parkinson’s disease [200]. Exogenous H_2_S reversibly increases intracellular calcium in microglial cells by releasing it from intracellular stores and facilitating its release through the plasma membrane [201]. Since H_2_S, like other gas transmitters, is capable of rapid diffusion, it is assumed that it may play an important role in the activation of vast populations of microglyocytes, increasing intracellular calcium levels in neighboring cells.

CBS-immunoreactive cells were detected in the periventricular region of the *medulla oblongata*, ventral and lateral zones of the carp cerebellum. The size of these cells, their location in the brain, and their relationship with H_2_S-producing neurons indicate that H_2_S-producing glia are present in the periventricular zone of the brain [202]. Since it has been established [203] that some neurotransmitters localized in the progenitor cells of the periventricular region of the brain can act as regulators of homeostatic neurogenesis, the assumption that H_2_S, like NO, can also act as a regulator of postnatal neurogenesis seems quite appropriate [192,203].

Studies have shown that neurons begin to secrete characteristic signaling molecules shortly after their formation from progenitor cells and well before the formation of interneuronal connections and the onset of synaptogenesis [204]. These molecules can include neuropeptides, enzymes for the synthesis of “classical” neurotransmitters and NO, transmembrane and vesicular transporters. Most of the signaling molecules are involved in the autocrine and paracrine regulation of target neuron differentiation, acting as morphogenetic or transcription factors [203]. It has been shown that NO plays the role of a signaling agent regulating the directed growth of axons and dendrites, as well as the migration of differentiating neurons [205]. In mammals, the activity of signaling molecules is limited to certain periods of ontogenesis, during which they exert a long-term morphogenetic effect on target neuron differentiation and the expression of specific phenotypes [206]. In fish, the postnatal neuro- and gliogenesis in the periventricular region continues into adulthood [149]. Previous studies have shown the presence of NADPH-positive and NOS-immunoreactive cells in the periventricular brain regions of juvenile *Oncorhynchus masou*. In Cypriniformes, NADPH-NOS activity is not detected in the periventricular region; apparently, H_2_S can act as a signaling molecule in this region of the carp brain [206].

The effects of exogenous cysteine and the slow-release hydrogen sulfide donor NaHS increase, and the administration of CSE and CBS inhibitors reduces the volume of cerebral infarction caused by unilateral closure of the middle cerebral artery. High cysteine levels associated with elevated H_2_S levels negatively affect the condition of patients with ischemic shock [207].

However, H_2_S may also exhibit protective properties for neurons. In particular, H_2_S protects neurons to a certain extent from the neurotoxic effects of glutamate. Increased glutamate production is observed in brain ischemia, epileptic seizures, and trauma. The neurotoxic effect of an excess of this transmitter usually manifests under conditions of prolonged activation of its receptors. However, glutamate can also cause oxytosis of neurons, regardless of the effect on the receptors of this transmitter. This damage mechanism is based on inhibition of cysteine transport into neurons. Extracellular glutamate blocks metabolism, leading to a deficiency of intracellular cysteine and suppression of glutathione synthesis; this condition makes the cell more sensitive to oxidative stress. NaHS increases the intracellular concentration of reduced glutathione and increases the concentration of cysteine and γ-glutamylcysteine (a precursor of glutathione) in rat cerebral cortex neurons in vitro [185].

Stimulation of afferent sensory nerves can cause inflammatory processes associated with the release of substance P, neurokinin-A, and calcitonin gene-related peptide (CGRP). These mediators, released in the cardiovascular and respiratory systems, induce a series of inflammatory responses, which include vasodilation, bronchoconstriction, mucus secretion, and plasma protein release, leading to edema. NaHS, like capsaicin, intensifies the release of substance P and CGRP from sensory nerves in the airways of guinea pigs [208]. Interestingly, intraperitoneal injection of NaHS into healthy mice causes a significant inflammatory response, accompanied by an increase in the concentration of substance P, proinflammatory cytokines, TNF-α and IL-1b [208,209]. These effects were eliminated by specific antagonists of substance P receptors (NK1, CP-96,345). The inflammatory effect of H_2_S was removed by capsazerin and was not detected in mice with substance P and neurokinin-A deficiency [209,210]. These data indicate that H_2_S can independently induce neurogenic inflammation even in the absence of other damaging factors.

## 13. Conclusions and Future Perspectives

This review summarizes the expanding body of data on adult neurogenesis in various vertebrate species, with a focus on teleost fish and mammals. Teleost fish serve as exceptional models for studying the dynamics of the cell cycle and the functions of adult neural stem and progenitor cells (aNSPCs) throughout the CNS. The lifelong presence of proliferating aNSPCs in various brain niches, along with their neuro-regenerative ability after damage to the brain and spinal cord, makes teleost fish extremely attractive for study. The analysis of early postnatal development and age-related changes in the organization of constitutive and post-traumatic processes, as well as the involvement of various regeneration-associated factors, can help fill gaps in current understanding of telencephalon development in juvenile salmonids, taking into account data on fetalization.

In all animal species studied to date, neuronal proliferation occurs throughout life. In birds, proliferation zones are most often located along the walls of the lateral ventricle of the forebrain and produce new neurons in the dorsal nuclei of the telencephalon in the region of the nidopallium (the highest vocal center), the hippocampus, and striatal structures (the paraolfactory lobe containing the nucleus of the vagus nerve). A fundamental difference with other species is observed in teleost fish, where adult neurogenesis takes place in all parts of the brain, as in other vertebrates; however, it is concentrated at local points, which indicates the existence of some local events regulating the processes of neurogenesis.

The sources of new neurons in adult mammals are NSCs, whose main properties are proliferative ability and multipotency. Currently, accurate identification of endogenous adult NSCs and their verification by existing methods remains challenging.

Currently, various molecular markers are used to study the properties of aNSPCs, which, in combination with PCNA and BrdU proliferation markers, make it possible to classify the precursors as glial or non-glial and determine the rate of proliferation. Glial-type precursors express GFAP, BLBP, and vimentin and have the morphology of glial cells. Studies on *D. rerio* indicate the existence of factors that keep stem cells in the ventricles. BrdU-labeled cells persist in the periventricular region for several weeks. Since such cells were found in two-year-old individuals, they probably persist at a later age. These data indicate that some progenitor cells in the adult telencephalon of *D. rerio* form a slowly proliferating, self-renewing population. It should be noted that these label-preserving cells express glial factors similar to mammalian NSCs.

The study of adult fish neurogenesis is relevant for several reasons. Firstly, NSCs are found in all regions of the fish brain, and currently, 16 neurogenic niches located along the anterior–posterior axis of the brain have been identified in zebrafish. Secondly, unlike mammals, the process of neuronal regeneration in the brain of fish is successful; after a traumatic injury, the neuronal structure is restored in the post-traumatic space.

Unlike mammals, in which an astrocytic scar is formed, in fish, neuronal conduction is restored in the area of damage, i.e., dead neurons are replaced by new ones. This is ensured by a number of adaptive mechanisms in the fish brain, in particular, enhanced synthesis of glutamine synthetase, aromatase B, H_2_S, and other factors. The presence of these regeneration-associated factors contributes to the rapid and successful recovery of nervous tissue after traumatic injury, resulting in the functional restoration of the neural structure, rather than the formation of a glial scar, as in mammals. The therapeutic applicability of factors promoting regeneration, in particular, GS and H_2_S, is based on their strong antioxidant properties, primarily due to the restoration of intracellular glutathione, which mitigates the effects of oxidative stress.

It is important to note that the effects of many regeneration-associated factors in the fish brain go beyond the maintenance of redox balance and also include the modulation of neuroinflammatory processes (aromatase B), neurotransmitter systems (TH, NADPH-diaphorase), mitochondrial functions, and neurotrophic support. It has now been established that these systems in the brains of juvenile salmon are markers of adult-type precursors, i.e., clarifying the functional properties of these enzyme systems in aNSPCs is a difficult and urgent task for future research. In particular, the presence of a large number of mitochondria in aNSPCs may indicate both glutathione-dependent and glutathione-independent effects. The stimulation of antioxidant protection through these pathways and the regulation of sulfur metabolism and its derivatives in mitochondria make it possible to consider aNSPCs of juvenile salmon as cells characterized by increased resistance to oxidative and mitochondrial dysfunction.

It can be assumed that the presence of a large number of aNSPCs in the brain of juvenile salmon is associated with a decrease in neuroinflammation, inhibition of critical inflammatory mediators, in particular iNOS, which has not been detected in the salmon brain, as well as pro-inflammatory cytokines. Recent studies have shown the presence of resident and activated microglia in the brain of juvenile salmon *O. keta* [211], whose functions can be modulated by the systems described above. Given the critical role of these systems in disorders in which neuroinflammation is a central etiological factor, such as Alzheimer’s disease and Parkinson’s disease, the study of neuroimmune relationships in the fish brain also seems relevant.

In addition, various regeneration-associated factors have significant effects on neurotransmitter systems, especially on the glutamatergic and dopaminergic pathways. Given the widespread distribution of GS [15] and TH in the brain of salmonids [149], which is closely related to the metabolism of glutamate and dopamine, as well as the expression of these enzymes in the periventricular precursors of the telencephalon, tectum, cerebellum, *medulla oblongata*, and spinal cord, it is safe to assert a significant influence of these factors on the synaptic release of glutamate and the maintenance of excitatory-inhibitory balance and neuroprotection.

This mechanism may prove useful in the development of new therapeutic strategies for conditions such as addiction, schizophrenia, and mood disorders. The influence of TH-expressing precursors on dopaminergic transmission—through redox regulation, modulation of dopamine transporters, and effects on dopamine metabolism—may hold promise for the development of novel treatments for Parkinson’s disease and substance use disorders.

In addition to modulating classical neurotransmitters, regeneration-associated factors can influence the expression of neurotrophic factors, epigenetic regulation, and endocrine signaling (for example, modulation of the hypothalamic-pituitary axis). These extensive neuromodulating effects indicate the potential of regeneration-associated factors of the fish brain as a promising tool for the treatment of complex multifactorial CNS diseases. Considering the wide range of mechanisms of action of regeneration-associated factors of the fish brain, the prospects for the treatment of multimodal disorders of the human CNS seem particularly promising.

Nevertheless, several significant gaps remain to be addressed in future studies, particularly regarding the enhancement of bioavailability and CNS penetration of these factors through improved delivery methods. In addition, it is essential to investigate the synergistic effects of various regeneration-associated factors, including their neuromodulatory, neuroprotective, immunomodulatory, and neuroendocrine properties, which may reveal new therapeutic targets. Expanding research to less-studied neurotransmitter systems and neuro-immune and neuroprotective interactions may further uncover novel therapeutic applications.

## Data Availability

No new data were created or analyzed in this study. Data sharing is not applicable to this article.

## Abbreviations

aNSC	adult neural stem cells
aNSPCs	adult neural stem progenitor cells
BLBP	brain lipid binding protein
BrdU	2′-deoxy-5-bromodeoxyuridine
CBS	Cystathionine-β-synthase
CNN	constitutive neurogenic niche
CNS	central nervous system
Dc	doublecortin
Dd	Dorsal pallial zone
Dl	Lateral pallial zone
Dm	Medial pallial zone
GABA	Gamma-aminobutyric acid
GFAP	Glial fibrillary acidic protein
GS	glutamine synthetase
HuCD	neuronal protein
ICH	immunohistochemistry
ip	immunopositive cells
MG	methyl green
NADPH	Nicotinamide adenine dinucleotide phosphate phosphorase
NECs	neuroepithelial cells
NMDA	ionotropic glutamate receptor
NOS	nitric oxide synthase
NPC	neural progenitor cells
NSCs	neural stem cells
OLB	olfactory bulb
PCNA	Nuclear antigen of proliferating cells
PVZ	periventricular zone
RG	radial glia
RMS	rostral migration stream
RNN	reactive neurogenic niche
SGZ	subgranular zone
SVZ	subventricular zone
TBI	traumatic brain injury
TF	transcription factor
TH	tyrosine hydroxylase
Vd	dorsal subpallial zone
Vim	vimentin
Vl	lateral subpallial zone
Vv	ventral subpallial zone
